# Modulation of Suppressive Activity and Proliferation of Human Regulatory T Cells by Splice-Switching Oligonucleotides Targeting FoxP3 Pre-mRNA

**DOI:** 10.3390/cells13010077

**Published:** 2023-12-29

**Authors:** Varvara G. Blinova, Yulia A. Gladilina, Anna A. Abramova, Daria D. Eliseeva, Valentina V. Vtorushina, Anastasia N. Shishparenok, Dmitry D. Zhdanov

**Affiliations:** 1 Laboratory of Medical Biotechnology, Institute of Biomedical Chemistry, Pogodinskaya st. 10/8, 119121 Moscow, Russia; varya.blinova@list.ru (V.G.B.); leonova_y@mail.ru (Y.A.G.); abramova.neurology@gmail.com (A.A.A.); a.shishparyonok@yandex.ru (A.N.S.); 2 Research Center of Neurology, Volokolamskoe Shosse, 80, 125367 Moscow, Russia; ddeliseeva@gmail.com; 3 National Medical Research Center for Obstetrics, Gynecology and Perinatology Named after Academician V.I. Kulakov of Ministry of Healthcare of the Russian Federation, Laboratory of Clinical Immunology, Academician Oparin st. 4, 117997 Moscow, Russia; vtorushina@inbox.ru; 4 Department of Biochemistry, People’s Friendship University of Russia Named after Patrice Lumumba (RUDN University), Miklukho-Maklaya st. 6, 117198 Moscow, Russia

**Keywords:** regulatory T cells, alternative splicing, FoxP3, suppressive activity, splicing-switching oligonucleotides, multiple sclerosis

## Abstract

The maturation, development, and function of regulatory T cells (Tregs) are under the control of the crucial transcription factor Forkhead Box Protein 3 (FoxP3). Through alternative splicing, the human FoxP3 gene produces four different splice variants: a full-length variant (FL) and truncated variants with deletions of each of exons 2 (∆2 variant) or 7 (∆7 variant) or a deletion of both exons (∆2∆7 variant). Their involvement in the biology of Tregs as well as their association with autoimmune diseases remains to be clarified. The aim of this work was to induce a single FoxP3 splice variant in human Tregs by splice switching oligonucleotides and to monitor their phenotype and proliferative and suppressive activity. We demonstrated that Tregs from peripheral blood from patients with multiple sclerosis preferentially expressed truncated splice variants, while the FL variant was the major variant in healthy donors. Tregs with induced expression of truncated FoxP3 splice variants demonstrated lower suppressive activity than those expressing FL variants. Reduced suppression was associated with the decreased expression of Treg-associated suppressive surface molecules and the production of cytokines. The deletion of exons 2 and/or 7 also reduced the cell proliferation rate. The results of this study show an association between FoxP3 splice variants and Treg function and proliferation. The modulation of Treg suppressive activity by the induction of the FoxP3 FL variant can become a promising strategy for regenerative immunotherapy.

## 1. Introduction

Regulatory T cells CD4^+^FOXP3^+^ (Tregs) control the immune response to autoantigens and play a key role in the development of autoimmune diseases [1,2]. In patients with some autoimmune diseases (multiple sclerosis (MS), Sjögren’s syndrome, psoriasis, etc.), a decrease in the number of Tregs and/or a lack of their ability to suppress autoactivated lymphocytes was revealed [3,4,5,6]. The reasons why these deviations develop are not fully understood. The transcription factor Forkhead Box Protein 3 (FoxP3) is a “master protein” that regulates differentiation and maturation as well as the intensity of proliferation and suppressor activity of Tregs [7,8].

Alternative splicing (AS) is a variant of messenger RNA (mRNA) splicing in which several types of mature mRNA can be formed during gene expression based on the same primary transcript (pre-mRNA). This is achieved by selective incorporation of exons (or parts of exons) of the primary transcript into the mature mRNA [9]. According to GenBank, four isoforms of the FoxP3 protein resulting from alternative splicing of its pre-mRNA have been described: a full-length variant (FL), variants with deletions of each of exons 2 (∆2 variant) or 7 (∆7 variant), and a deletion of both exons (∆2∆7 variant). It should be clarified that there are differences in the numbering of these exons in the literature, and exons 2 and 7 (considering only protein-coding exons) in some sources are designated 3 and 8, respectively, considering the noncoding exon as the first [10,11].

Although several studies have demonstrated the involvement of FoxP3 splice variants in the biology of Tregs [10,12,13,14], a comparative investigation of the influence of these splice variants on Treg proliferation, immunophenotype, and suppressive activity remains to be performed. Moreover, the roles and associations of FoxP3 splice variants with the onset and progression of autoimmune diseases have never been studied before.

Splice switching oligonucleotides (SSOs) are short synthetic single-stranded nucleotides that selectively bind to a target pre-mRNA through Watson-Crick base pairing [15,16]. Binding to target pre-mRNA creates a steric block to the binding of splicing factors and alters the recognition of splice sites by regulatory splicing proteins or the spliceosome. This leads to selective inclusion or deletion of the exon in the mature mRNA transcript. Thus, SSOs have become a powerful tool for the selective induction of splice variants in cells for biomedical purposes.

In this work, we demonstrated changes in the proportion of FoxP3 splice variants in peripheral blood Tregs from patients with multiple sclerosis in comparison to healthy donors. The selective induction of each of the four FoxP3 splice variants by SSOs had an effect on Treg proliferation and suppressive activity. 

## 2. Materials and Methods

### 2.1. Patients, Healthy Donors, and Blood Sampling

All procedures in studies involving human subjects were carried out in compliance with the institutional and national research committee’s ethical standards, as well as the 1964 Helsinki statement and its later updates or comparable ethical standards. This study was approved by the Local Ethics Committee of the Scientific Center for Neurology (no. 12-3 of 28 December 2020). Study subjects were recruited in the outpatient department of the Research Center of Neurology (Moscow, Russian Federation). All patients gave written informed consent to participate in this study.

Only adult patients (aged 18 and older) were recruited into this study. Patients with acute and chronic infectious diseases and/or a history of neuroinfectious diseases, those with a history of cardiovascular disease, diabetes mellitus, malignancies, and active smokers were excluded from this study. Demographic, epidemiological, and clinical data were collected by interviewing study subjects and via electronic health records. Prior to blood sampling, each patient underwent detailed physical and neurological examinations. The main group consisted of 20 patients diagnosed with MS. The diagnosis of MS was established using the McDonald criteria (2017) [17]. All patients were clinically evaluated using the Expanded Disability Status Scale (EDSS) [18].

The control group of healthy donors (HD) consisted of 20 healthy subjects who were age- and sex-matched to the MS group. A physical examination and neurological assessment were performed for all subjects recruited into the healthy control group to rule out any underlying neurological conditions.

Blood samples were obtained via venipuncture in Vacuette K_3_EDTA tubes (Greiner Bio-One, Kremsmünster, Austria).

### 2.2. Detection of Tregs and Treg-Associated Cellular Markers in Peripheral Blood Samples 

MACS Quant Analyzer 10 flow cytometer and MACSQuantify™ Software 3.0 (both Miltenyi Biotec, Bergisch Gladbach, Germany) were used for flow cytometry analysis. The detection of Tregs in the peripheral blood samples was performed using immunofluorescence staining of Treg cellular antigens. Cells were stained with Treg Surface Marker Analysis Cocktail (CD45-VioBlue, CD4-FITC, CD25-APC, CD127-PE; #130-096-082, Miltenyi Biotec, Bergisch Gladbach, Germany) according to the manufacturer’s protocol. The gating strategy for Treg detection for MS patients and HDs is shown in Figure S1 in the Supplementary File.

To analyze the expression of Treg-associated surface markers, transfected cells were labeled with one of the following antibodies: anti-human CD4–FITC (#130-113-775), anti-human CD25–APC (#130-123-832), anti-human CD127–FITC (#130-113-409), anti-human CD152–APC (#130-123-812), anti-human CD39–FITC (#130-125-094), or anti-human CD223–APC (#130-119-567), all from Miltenyi Biotec (Bergisch Gladbach, Germany), and analyzed by flow cytometry. The expression was determined by the mean fluorescence intensity (MFI).

For the detection of nuclear markers FoxP3 and Helios, cells were fixed and permeabilized using a Transcription Factor Buffer Set (#562574, BD Pharmingen, East Rutherford, NJ), labeled with anti-human FoxP3-PE (#130-125-587) or anti-human Helios-PE antibodies (#130-104-033). 

To measure the intracellular location of granzymes, Tregs were stimulated with the Stimulation Cocktail + Protein Transport Inhibitor (#00-4975-03, eBioscience Inc., San Diego, CA, USA) for 4 h in a CO_2_ incubator. Control cells were incubated only with the protein transport inhibitor (#00-4980-93, eBioscience Inc., San Diego, CA, USA). After stimulation, the cells were fixed and permeabilized as described above and labeled with anti-human granzyme A-PE (#130-123-973), anti-human/mouse/rat granzyme B-PE (#130-116-654), or anti-human perforin-FITC (#130-096-668), all from Miltenyi Biotec (Bergisch Gladbach, Germany), and analyzed by flow cytometry.

### 2.3. Treg Purification and Ex Vivo Expansion

Fresh peripheral blood mononuclear cells (PBMCs) were obtained using Lympholite-H (Cedarlane, Burlington, Ontario, Canada) by density gradient centrifugation. Tregs were isolated using the CD4^+^CD25^+^ Regulatory T-Cell Isolation Kit (Miltenyi Biotec, Bergisch Gladbach, Germany, #130-091-301) according to the manufacturer’s protocol. The purity of the obtained Tregs was monitored by flow cytometry using a Treg Surface Marker Analysis Cocktail (Miltenyi Biotec, Bergisch Gladbach, Germany, #130-096-082) and by the detection of the nuclear markers FoxP3 and Helios described above. 

Expansion ex vivo was performed as described elsewhere [19]. Briefly, Tregs were purified from PBMCs, seeded at 5 × 10^4^ cells/mL, and cultured in 25 cm^2^ flasks in RPMI-1640 cell medium (Thermo Fisher Scientific Inc., Waltham, MA, USA) supplemented with 10% FBS (Fetal Serum Bovine, Thermo Fisher Scientific Inc.), 2 μg/mL anti-CD28 antibodies (eBiosciences, San Diego, CA, USA), 5 μg/mL anti-CD3 monoclonal antibodies (MedBioSpectr, Moscow, Russia), and 100 U/mL rHu IL-2 (R&D Systems, Minneapolis, MN, USA). Every 3 days of expansion, the cells were split to 5 × 10^5^ cells/mL in fresh full medium and cultivated for up to 15 days. The number of proliferating cells and cell viability were measured every 3 days using a dye exclusion test with Trypan Blue Solution and the Cell Viability Analyzer Vi-Cell XR (Beckman Coulter, Brea, CA, USA) according to the manufacturer’s protocol. The frequency of cell cycles per day (*f*) was determined by the equation *N_t_ = N*_0_*2^tf^*, where *N_t_* is the number of cells at time *t* (day) and *N*_0_ is the initial number of cells [20,21]. Proliferating cells were photographed using a Biomed 3I inverted microscope (Biomed, Saint Petersburg, Russia) within four days of cultivation.

### 2.4. RNA Isolation and Real-Time RT-PCR

The procedure was carried out in accordance with a previously described protocol [22], using the primers listed in Table S1 in the Supplementary File. The data of the obtained mRNA level were normalized in relation to the mean RNA levels of 18S, glyceraldehyde-3-phosphate dehydrogenase (GAPDH), and beta-actin genes, which were used as references. Utilizing DTPrime5 software, the relative RNA concentration was calculated (DNA Technology, Protvino, Russia).

### 2.5. Cell Transfection with Splice-Switching Oligonucleotides

The transfection of expanded Tregs with 36-mer splice-switching oligonucleotide (SSO) base pairing with FoxP3 pre-mRNA or control 36-mer oligonucleotides was performed using Lipofectamine 2000 (Invitrogen, Grand Island, NY, USA) according to the manufacturer’s protocol. They could base-pair regulatory sequences and induce deletion or retention of exon 2 or exon 7 by blocking splicing regulatory proteins from binding to their sensitive *cis*-elements. The nucleotides (custom synthesized by Evrogen, Moscow, Russia) were uniformly modified with 2′-O-(2-methoxy) ethyl sugars (2′MOE), a phosphorothioate backbone, and 5′-methyl cytosine as described in [21,23] and conjugated with Cy3 or Cy5.5 dye. The SSO sequences are provided in Table 1.

### 2.6. Western Blotting

Detection of the exon 2-containing splice variant at the protein level was performed by Western blotting according to a previously described protocol [24]. Purified anti-mouse/rat/human FoxP3 antibody (clone 150D, #320001), which is an exon 2-specific antibody, and purified anti-human FoxP3 antibody (clone 259D, #320201, both from BioLegend, San Diego, CA, USA), which recognizes an epitope after exon 2 common for all splice variants, were used as primary antibodies. Glyceraldehyde-3-phosphate dehydrogenase (GAPDH) was used as a reference loading control and was detected by anti-GAPDH (clone 6C5, #ab8245, Abcam, Cambridge, MA, USA). Membranes were visualized using the Super Signal chemiluminescent kit (Pierce Biotechnology, Rockford, IL, USA).

A BLAST search for SSO target sequences revealed no other perfect sequence matches within the human genome.

To determine the efficiency of transfection, Cy3-positive and Cy5.5-positive cells were counted by flow cytometry with a MACS Quant Analyzer 10 (Miltenyi Biotec, Bergisch Gladbach, Germany). The cellular load of the SSOs or control oligonucleotides was determined by the MFI of Cy3-positive and Cy5.5-positive cells.

Transfected cells were cultivated for 96 h. The number of proliferating cells and cell viability were measured daily, as described above. Cell imaging was assessed using an inverted microscope, Biomed 3I (Biomed-Service, Moscow, Russia), at the end of cultivation time.

To measure cell proliferation, transfected Tregs were labeled with the vital dye carboxyfluorescein succinimidyl ester (CFSE, Life Technologies, Carlsbad, CA, USA) and analyzed by flow cytometry every 24 h. The number of proliferating cells was estimated daily by the number of cells with a reduced CFSE signal using a flow cytometry count [21,25].

To measure the viability of transfected cells within the time of cultivation, 10^4^ cells were resuspended in 100 μL of PBS and stained with propidium iodide (PI, 10 μg/mL, Helicon, Moscow, Russia) each 24 h. The percent of PI-positive cells was counted by flow cytometry [26].

### 2.7. Suppression Assay

To study Treg suppressive activity, mixed lymphocyte reaction (MLR) was performed in 96-well round-bottom plates (Sigma-Aldrich, St. Louis, MO, USA) in a final volume of 200 μL/well. Responder CD4^+^CD25^−^ T cells (target cells) were obtained from the negative fraction during CD4^+^CD25^+^ cell purification and then labeled with the vital dye (CFSE, Life Technologies, Carlsbad, CA, USA). Labeled cells were grown in triplicate at 5 × 10^4^ cells/well with allogeneic mitomycin C-treated PBMCs (Kyowa Hakko Kirin, Tokyo, Japan) with or without Tregs in the presence of 5 g/mL anti-CD3 antibodies for 5 days at 37 °C and 5% CO_2_. To detect suppression, CFSE-labeled target cells were analyzed on a MACS Quant analyzer 10. The proportion of proliferating cells was determined by the number of cells with a reduced CFSE signal.

### 2.8. Inhibition of Telomerase

The ability of the Tregs to inhibit telomerase in the target cells was tested in a coculture procedure, as described in previous studies [27,28]. Transfected Tregs at concentration 5 × 10^4^ cells/well were co-cultivated with target CD4^+^CD25^−^ T cells at a 1 to 3 ratio in a 3 μm porous membrane-insert system Millicell Culture Inserts, (Millipore, Bedford, MA, USA) in 24-well flat-bottom plates (Nunc, Roskilde, Denmark) in a final volume of 1000 μL/well in quadruplicate. Cells were fed by replacing the complete medium supplemented with IL-2 and antibodies every 4 days. Telomerase activity in target cells was determined using the Telomeric Repeat Amplification Protocol (TRAP). Briefly, cells were lysed in 10 mM Tris-HCl, pH 7.5, 1 mM MgCl2, 1 mM EGTA, 0.1 mM PMSF, 5 mM 2-Mercaptoethanol, 0.5% CHAPS, and 10% glycerol (all from Sigma-Aldrich) and centrifuged for 30 min at 12,000× *g*. Elongation of the oligonucleotide substrate TS-primer (Telomerase Substrate Primer) (5′-AATCCGTCGAGCAGAGTT-3′) and subsequent amplification were conducted in a 30 μL reaction mixture containing 67 mM Tris-HCl, pH 8.8, 16.6 mM (NH_4_). _2_SO_4_, 0.01% Tween-20, 1.5 mM MgCl_2_, 1 mM EGTA (all from Sigma-Aldrich), 0.25 mM dNTPs (Evrogen, Moscow, Russia), and 2 μL of cell lysate (equivalent to 2000 cells). Elongation was performed for 30 min at 37 °C, followed by 10 min at 96 °C for telomerase inactivation. Then, 0.1 μL of CX-primer (Copy Extended primer) (5′-CCCTTACCCTTACCCTTACCCTAA-3′) and 2.5 units of Taq polymerase were added to the elongation mixture, followed by PCR using the following reaction conditions: (1) 94 °C for 5 min; (2) 30 cycles of 94 °C for 30 sec, 50 °C for 30 sec, and 72 °C for 40 sec; and (3) 72 °C for 5 min. PCR product visualization was performed by 12% non-denaturing PAAG electrophoresis with TBE buffer. Ten microliters of samples were added to each well from a gel comb. The gels were stained with SYBR Green I (Invitrogen, Grand Island, NY, USA), photographed under UV light in a ChemiDoc™ XRS imaging system, and analyzed with GelAnalyzer 2010a.

### 2.9. Determination of Cytokines by Bio-Plex Assay

Determination of the concentration of IL-10, IL-12 (p40), IL-12 (p70), IL-19, IL-20, IL-22, IL-26, IL-27 (p28), IL-28A/IFN-λ2, IL-29/IFN-λ1, and IL-35 was carried out by the multiplex method using the standard 12-Plex Bio-Plex Pro ™ Human Treg Cytokine Panel, 12-Plex (#171AL003M, Bio-Rad, Hercules, CA, USA) on the Bio-Plex 200 System immunoanalyzer (Bio-Rad, Hercules, CA, USA) and subsequent processing of the obtained results using the Bio-Plex Manager 6.0 Properties application (Bio-Rad, Hercules, CA, USA).

### 2.10. Statistics

Statistical tests were performed in SPSS 25 software (IBM SPSS Statistics, Armonk, NY, USA). Statistical differences were analyzed by the Mann–Whitney U test or Kruskal–Wallis test. As needed, the Student’s *t* test and its Bonferroni modification were used. The results are presented as the mean ± standard deviation (SD) from individual determinations with at least three replicates. *p* values were considered significant. * *p* < 0.05, ** *p* < 0.01, *** *p* < 0.001. 

## 3. Results

### 3.1. Study Participants

The expression of FoxP3 splice variants was studied in Tregs isolated from 20 patients with diagnosed MS and from 20 healthy donors.

In Table 2, we summarized the main demographic characteristics of all study groups.

The main clinical characteristics of the MS group are provided in Table 3. All data are presented as the mean ± SD, with a 95% confidence interval.

Among all patients in the MS group, relapsing-remitting MS (RRMS) was observed in 16 (80%) cases, and secondary progressive MS (SPMS) was observed in 4 (20%) cases. Three patients with SPMS demonstrated signs of disease activity (relapses and/or new demyelinating lesions on brain and spinal cord MRI) 12 months prior to sampling and, thus, could be classified as having active SPMS.

In five MS cases, a lumbar puncture was never performed. Among the remaining 15 subjects, 13 patients (88.2%) had intrathecal oligoclonal IgG synthesis.

At the time of enrollment, 11 MS patients (55%) were in the remission phase, while 9 patients (45%) experienced disease relapse, which was defined as the occurrence of new neurological symptoms or worsening of old symptoms persisting for more than 24 h and a follow-up time period of three months. Patients who experienced disease relapse had not received any relapse treatment before blood sampling.

Among all MS patients, 15 subjects (75%) never received any disease-modifying medications (disease-modifying therapy (DMT)-naïve patients). Four patients (20%) were treated with DMTs at the moment of enrollment (two with interferon beta-1b, one with interferon beta-1a, and one with teriflunomide), with a mean therapy duration of 3.9 ± 2.0 years. One patient received teriflunomide for one month, six months prior to study enrollment, followed by the standard washout procedure. Finally, two patients were treated with glatiramer acetate for two and three years, respectively, but discontinued therapy more than two years before this study.

### 3.2. Expression of FoxP3 Splice Variants in Tregs from MS Patients

**Figure 1:**
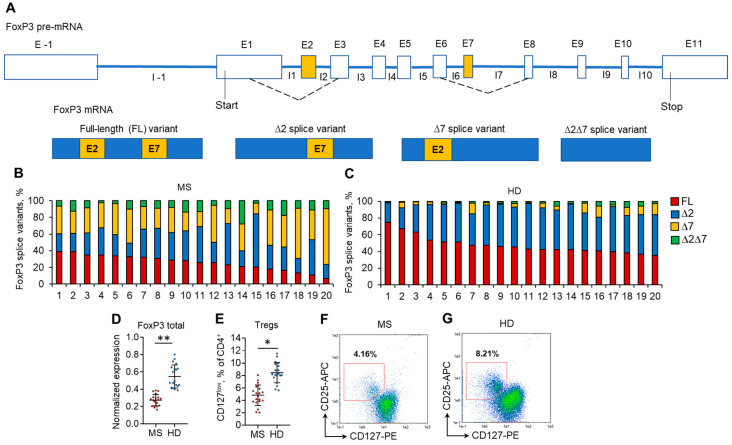
Changes in the expression of FoxP3 splice variants are associated with a reduced proportion of Tregs in the peripheral blood of MS patients. (**A**) Schematic presentation of FoxP3 pre-mRNA AS resulting in the formation of four types of mature splice variant mRNAs: the full-length (FL) variant includes both exon 2 and exon 7; the ∆2 splice variant lacks exon 2 and includes exon 7; the ∆7 splice variant lacks exon 7 and includes exon 2; and the ∆2∆7 lacks both exon 2 and exon 7. Proportions of FoxP3 mRNA splice variants in Tregs isolated from the peripheral blood of (**B**) 20 MS patients and (**C**) 20 healthy donors (HD). The levels of investigated mRNAs were normalized to the mean expression of three reference genes: 18S, GAPH, and beta-actin. (**D**) The level of total FoxP3 mRNA demonstrates its reduced expression in Tregs from MS patients in comparison to HDs. (**E**) Reduced proportion of CD4^+^CD25^+^CD127^low^ Tregs in peripheral blood of MS determined by flow cytometry. Black horizontal lines indicate the median. n = 20 for both the MS and HD groups. * *p* ≤ 0.05; ** *p* ≤ 0.01 by the Mann–Whitney U test. Representative flow cytometry diagrams demonstrate a reduced proportion of Tregs in (**F**) MS patients in comparison to (**G**) HDs.

The mRNA levels of each of four FoxP3 splice variants, FL, ∆2, ∆7, and ∆2∆7 (Figure 1A), were measured by real-time RT–PCR. All four splice variants were detected in both MS and HD Tregs from peripheral blood (Figure 1B,C). However, significant changes were observed in the proportion between them. In the HD group, the predominant splice variants were FL (median 44.2%; mean 47.6 ± 10.4%) and ∆2 (median 46.7%; mean 44.2 ± 8.7%), while in MS patients, a substantial decrease in the proportion of FL variants (median 27.0%; mean 26.0 ± 8.9%; *p* ≤ 0.05 vs. HD group) was observed. The proportion of the ∆2 splice variant in MS patients decreased nonsignificantly (median 30.5%; mean 32.0 ± 11.9%; *p* > 0.05 vs. HD group, Figure S2 in the Supplementary File). We observed an increase in the proportion of ∆7 and ∆2∆7 splice variants in Tregs from MS patients. In Tregs from HD, ∆7 (median 3.6%, mean 5.5 ± 4.6%) and ∆2∆7 (median 2.2%, mean 2.8 ± 1.9%) splice variants were at very low proportions. However, in Tregs from MS patients, they were significantly induced: ∆7 (median 31.1%, mean 31.9 ± 10.8%; *p* ≤ 0.01 vs. HD group) and ∆2∆7 (median 9.3%, mean 10.1 ± 5.7%; *p* ≤ 0.05 vs. HD group).

The changes in the proportion of FoxP3 splice variants corresponded to the decreased expression of total FoxP3 in Tregs (Figure 1D) and decreased Treg percentage (Figure 1E–G, Figure S2, Tables S2 and S3 in the Supplementary file) in the peripheral blood of MS patients. 

The patients were clinically heterogeneous, so there were no correlations between FoxP3 splice variants and clinical manifestations.

### 3.3. Expansion Ex Vivo Does Not Lead to a Shift in the Expression of FoxP3 Splice Variants

**Figure 2:**
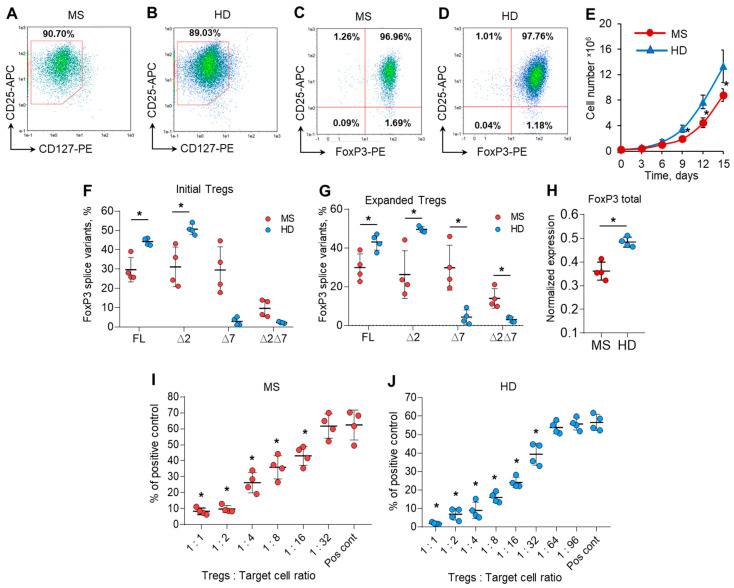
Ex vivo expansion has no effect on the level of FoxP3 mRNA splice variants. Expanded Tregs from MS patients demonstrated reduced proliferative and suppressive activities. Tregs isolated from the peripheral blood of MS patients and HDs were multiplied ex vivo for 15 days as described in the Materials and Methods. Representative flow cytometry diagrams demonstrate the homogeneity of expanded Tregs obtained from (**A**,**C**) MS patients and (**B**,**D**) HDs by the expression of CD127^low^ or FoxP3 in CD25^+^-positive cells. (**E**) Number of cells during 15 days of ex vivo expansion demonstrating reduced proliferative activity of cells from MS patients Real-time RT-PCR results demonstrate no changes in the proportion of FoxP3 slice variant mRNA levels in (**F**) initial Tregs and (**G**) expanded Tregs. (**H**) The level of total FoxP3 mRNA demonstrates its reduced expression in expanded Tregs of MS patients in comparison to that of HDs. The levels of investigated mRNAs were normalized to the mean expression of three reference genes: 18S, GAPDH, and beta-actin. The results of the mixed lymphocyte reaction test (MLR) demonstrate the reduced suppressive activity of expanded Tregs in (**I**) MS patients in comparison to (**J**) HDs. The results are shown as the percentage of proliferated cells measured by the reduction in the CFSE signal using flow cytometry. Representative flow cytometry proliferation diagrams for the MLR test are shown in Figure S5 in the Supplementary File. Pos. cont.—positive control, CD4^+^CD25^−^ cells cocultured with accessory cells. n = 4 for both the MS and HD groups. * *p* ≤ 0.05 by Mann–Whitney U test.

Very few cells could be obtained from the provided volume of blood, and the purpose of this part of the work was to obtain quantities of cells applicable for further study. Tregs were isolated from the peripheral blood of MS patients (patients 9, 10, 11, and 12 from Figure 1B) and HDs (donors 9, 10, 11, and 12 from Figure 1C) with a purity of 89.11–93.6% (Figure S3 in the Supplementary File) and multiplied ex vivo for 15 days. Expanded ex vivo cells demonstrated the immune profile of Tregs: 88.6–92.1% of cells had a CD127^low^ immunophenotype (Figure 2A,B), and 96.6–97.7% of them were FoxP3-positive (Figure 2C,D) for both the MS and HD groups. These results demonstrated the homogeneity of the population of ex vivo multiplicated Tregs (Figure S4 in the Supplementary File).

We observed a difference in the proliferation rate between Tregs from the MS and HD groups. Ex vivo cultivation allowed us to expand Tregs by 40.4–63.2 times their initial number for the HD group and 31.2–39.9 times for the MS group (Figure 2E).

We compared FoxP3 splice variant mRNA rates in isolated initial cells and those after expansion and found that the proportion of splice variants did not change in either the MS or HD groups (Figure 2F,G). The expression of total FoxP3 mRNA was also higher in expanded cells from the HD group than in those from the MS group (Figure 2H), which corresponds to the data for isolated Tregs shown in Figure 1D.

Our data demonstrated neither significant differences in the expression of cell markers measured by flow cytometry nor in the rate of FoxP3 splice variant expression between groups of cells isolated from blood or after cell expansion. Expanded cells were used for further experiments.

Tregs in MS patients have diminished suppressive activity, as shown in several studies [3,29,30]. We performed MLR to study the functional suppressive activity of 15-day ex vivo expanded Tregs from the MS and HD groups. Tregs from MS patients significantly suppressed the proliferation of autologous responder CD4^+^CD25^−^T cells at a ratio of 1:16 (Figure 2I and Figure S5 in the Supplementary File). Tregs from HDs were two times more active and could perform suppressive activity at a ratio of 1:32 (Figure 2J). Thus, we can conclude that Tregs expanded ex vivo in MS patients also exhibit reduced suppressive activity.

### 3.4. Modulation of Exon 2 and Exon 7 Alternative Splicing with Single Splice-Switching Oligonucleotides

**Figure 3:**
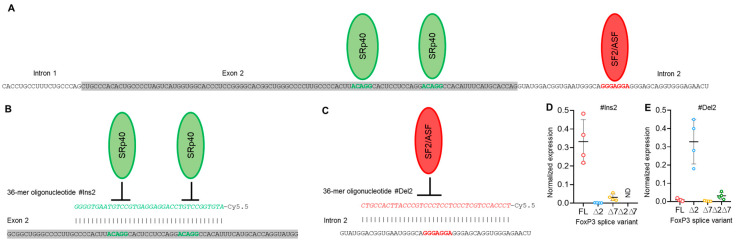
Modulation of FoxP3 pre-mRNA splicing with SSO targeting only exon 2 *cis*-elements does not allow us to obtain Tregs expressing a single splice variant. (**A**) Two splicing regulator proteins, SRp40 (shown as green ellipses), interact with their binding sites (shown in bold green font) within exon 2 and are responsible for the inhibition of exon 2 insertion in mature FoxP3 mRNA. The splicing regulator proteins SF2/ASF (shown as a red ellipse) interact with its binding site (shown in bold red font) within intron 2 and are responsible for the inhibition of exon 2 deletion from mature mRNA. (**B**) Treg transfection with #Ins2, a 36-mer-specific antisense SSO (presented in green italics font), blocks SRp40 from binding to its sensitive *cis*-elements and induces the insertion of exon 2 into the mature mRNA. (**C**) Treg transfection with #Del2, a 36-mer-specific antisense SSO (presented in red italics font), blocks SF2/ASF from binding to its sensitive *cis*-elements and induces the deletion of exon 2 from the mature mRNA. FoxP3 splice variant mRNA levels in cells 96 h after transfection with (**D**) #Ins2 or (**E**) #Del2 SSOs. The levels of investigated mRNAs were normalized to the mean expression of three reference genes: 18S, GAPDH, and beta-actin. N = 4. The results are shown as the mean ± SD. FL, full-length splice variant; ∆2, splice variant with deleted exon 2; ∆7, splice variant with deleted exon 7; ∆2∆7, splice variant with deleted both exon 2 and exon 7. ND, not detected.

**Figure 4:**
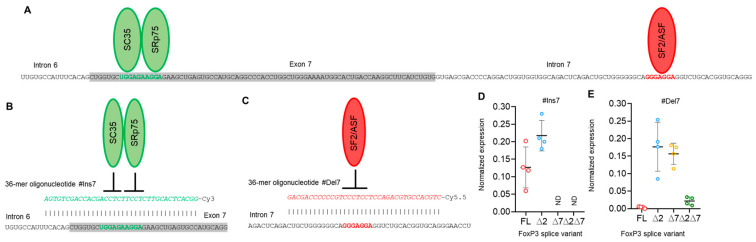
Modulation of FoxP3 pre-mRNA splicing with SSO targeting only exon 7 *cis*-elements does not allow us to obtain Tregs expressing a single splice variant. (**A**) Two splicing regulator proteins, SC35 and SRp75 (shown as green ellipses), interact with their binding sites (shown in bold green font) within exon 7 and are responsible for the inhibition of exon 7 insertion in mature FoxP3 mRNA. The splicing regulator proteins SF2/ASF (shown as a red ellipse) interact with its binding site (shown in bold red font) within intron 7 and are responsible for the inhibition of exon 7 deletion from mature mRNA. (**B**) Treg transfection with #Ins7, a 36-mer-specific antisense SSO (presented in green italics font), blocks both SC35 and SRp75 from binding to their sensitive *cis*-elements and induces the insertion of exon 7 into the mature mRNA. (**C**) Treg transfection with #Del7, a 36-mer-specific antisense SSO (presented in red italics font), blocks SF2/ASF from binding to its sensitive *cis*-elements and induces the deletion of exon 7 from the mature mRNA. FoxP3 splice variant mRNA levels in cells 96 h after transfection with (**D**) #Ins7 or (**E**) #Del7 SSOs. The levels of investigated mRNAs were normalized to the mean expression of three reference genes: 18S, GAPDH, and beta-actin. N = 4. The results are shown as the mean ± SD. FL, full-length splice variant; ∆2, splice variant with deleted exon 2; ∆7, splice variant with deleted exon 7; ∆2∆7, splice variant with deleted both exon 2 and exon 7. ND, not detected.

To modulate exon 2 and exon 7 alternative splicing in Tregs and to create a Treg cell line expressing only one of four possible FoxP3 splice variants, we created 36-mer splice-switching oligonucleotides (SSOs). They could base-pair to splicing regulatory sequences and induce deletion (Figure 3A,B) or retention of exon 2 or of exon 7 (Figure 4A,B) [31,32,33]. Tregs from four HDs (donors 9, 10, 11, and 12) were transfected with each single oligonucleotide, and the expression of FoxP3 splice variants was examined four days after transfection. Transfection efficiency was monitored every 24 h (Figure S6 in the Supplementary File). The use of each single SSO had an effect on target exon splicing. SSO #Ins2 could induce the inclusion of exon 2, and the predominant splice variant was FL (Figure 3 D), while the ∆2 variant was detected at a very low level. Splice variant ∆2∆7 was not detected at all. SSO #Del2 could induce the deletion of exon 2, and the predominant splice variant was ∆2 (Figure 3E). FL and ∆7 variants were expected to be expressed at very low levels. #Del2 SSO targeting exon 2 had no effect on the mRNA level of splice variant ∆2∆7.

SSO #Ins7 could induce the inclusion of exon 7, and the predominant splice variants were FL and ∆2 (Figure 4D), while variants with exon 7 deletions (∆7 and ∆2∆7) were not detected. SSO #Del7 could induce the deletion of exon 7 and, as expected, upregulate the amount of the ∆7 splice variant. The FL variant was detected in minor quantities (Figure 4E). Transfection with SSO targeting exon 7 had no effect on the mRNA level of splice variants with the deletion of exon 2, (∆2 & ∆2∆7 splice variants), whose expression did not change in comparison to nontransfected cells.

In Figure S7 in the Supplementary File, we provided the results of the expression of FoxP3 splice variants in nontransfected Tregs and Tregs transfected with control nonspecific SSO. 

### 3.5. Splice-Switching Oligonucleotides Targeting Both Exon 2 and Exon 7 on FoxP3 Pre-mRNA Induce Selective Expression of a Single Splice Variant

**Figure 5:**
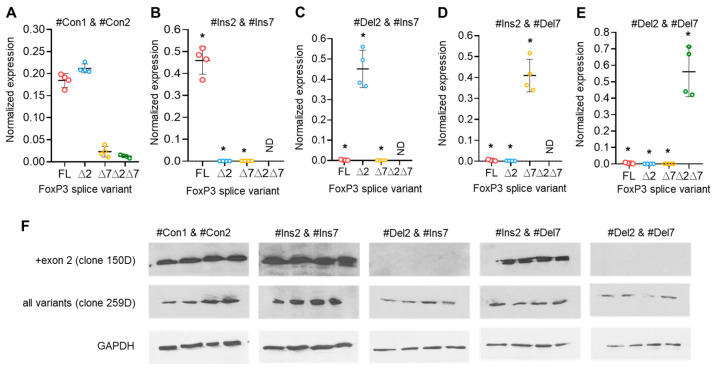
Modulation of FoxP3 pre-mRNA splicing with SSOs targeting both exon 2 and exon 7 *cis*-elements allowed us to obtain Tregs expressing a single splice variant. FoxP3 splice variant mRNA levels in Treg cells 96 h after transfection with (**A**) control nonspecific 36-mer nucleotides #Con1 & #Con2; (**B**) SSOs #Ins2 & #Ins7, which could induce the expression of the FL variant only; (**C**) SSOs #Del2 & #Ins7, which could induce the expression of the ∆2 splice variant only; (**D**) SSOs #Ins2 & #Del7, which could induce the expression of the ∆7 splice variant only; and (**E**) SSOs #Del2 & #Del7, which could induce the expression of the ∆2∆7 splice variant only. N = 4. The results are shown as the mean ± SD. * *p* ≤ 0.001 by Mann–Whitney U test. FL, full-length splice variant; ∆2, splice variant with deleted exon 2; ∆7, splice variant with deleted exon 7; ∆2∆7, splice variant with deleted both exon 2 and exon 7. ND, not detected. (**F**) Western blotting results demonstrate the induction of the ∆2 splice variant. Clone 150D is exon 2 specific, while clone 259D recognizes an epitope after exon 2 common for all FoxP3 splice variants.

The results of the previous experiment demonstrated that the use of a single SSO targeting only one of the exon 2 or exon 7 *cis*-regulating elements on FoxP3 pre-mRNA did not allow for the creation of a Treg cell line expressing a single splice variant. Transfection efficiency was monitored every 24 h (Figure S8 in the Supplementary File). We decided to transfect cells with a couple of SSOs that could selectively modulate exon 2 and 7 splicing. Transfection of cells with two SSOs responsible for the insertion of exon 2 (#Ins2) and exon 7 (#Ins7) resulted in the expression of the FL variant only, while the variants with deletions of the exons were expressed at very low levels or were not detected at all (Figure 5A,B). Tregs transfected with SSO-inducing exon 2 deletion (#Del2) and SSO #Ins7 showed expression of ∆2 only but not other splice variants (Figure 5C). The use of SSO #Ins2 and SSO #Del7, which are capable of inducing the deletion of exon 7, allowed us to obtain Tregs expressing the ∆7 variant but no other splice variants (Figure 5C). Two SSOs targeting the deletion of both exons (#Del2 and #Del7) resulted in the expression of only the shortest ∆2∆7 FoxP3 splice variant in transfected Tregs (Figure 5D). In Figure S9 in the Supplementary File, we provided the results of the expression of total FoxP3 in transfected Tregs, which demonstrated no changes after transfection. The results of FoxP3 splice variant mRNA quantification were confirmed by Western blotting (Figure 5F). Using each of two anti-FoxP3 antibodies (clone 259D recognizing a common epitope after exon 2 common for all variants and clone 150D specific for exon 2), we demonstrated the absence of the FoxP3 protein lacking exon 2 epitopes (i.e., ∆2 and ∆2∆7 splice variants).

The results of this experiment demonstrated that the approach based on the use of two SSOs selectively targeting exon 2 and exon 7 allowed us to obtain Tregs transiently expressing only one of four possible FoxP3 splice variants.

### 3.6. Selective Expression of FoxP3 Splice Variants Has an Effect on Treg Proliferation

The proliferative activity of Tregs transfected with a couple of SSOs and expressing a single FoxP3 splice variant was monitored every 24 h. The frequency of cell divisions per day in transfected cells is shown in Table 4.

**Figure 6:**
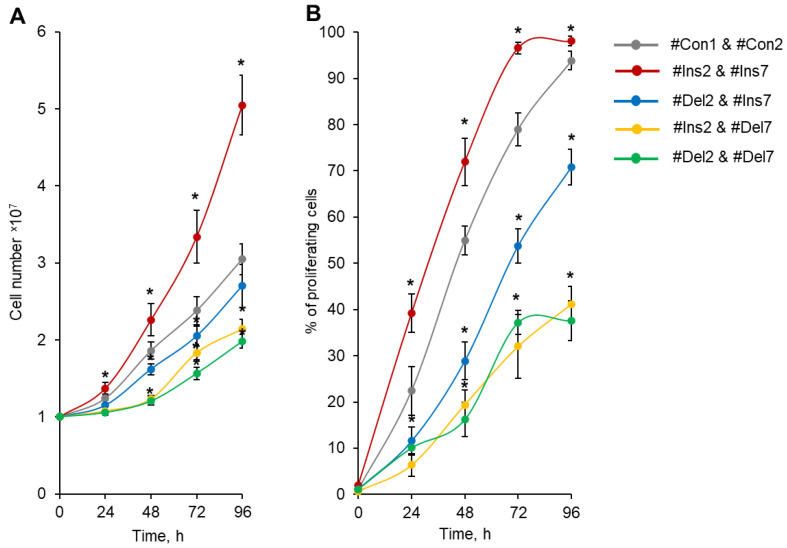
Tregs expressing only one FoxP3 splice variant demonstrated different proliferative activities. Tregs were transfected with each SSO, inducing the expression of only one of the FoxP3 splice variants, and expanded for 96 h. (**A**) Proliferative activity (number of cells) during 4 days of cultivation after transfection, demonstrating increased proliferative activity for cells expressing the FL variant and decreased proliferative activity in cells with exon deletions in splice variants. (**B**) Percentage of proliferating cells within four days of proliferation after transfection. n = 4. * *p* ≤ 0.05 vs. cells transfected with #Con1 & #Con2 oligonucleotides by the Mann–Whitney U test.

Total cell number calculation demonstrated that Tregs transfected with #Ins2 & #Ins7 SSOs (expressing only the FL splice variant) had an increased rate of proliferation, and 96 h after transfection, the number of cells was (5.05 ± 0.39) × 10^7^ in comparison to (3.05 ± 0.20) × 10^7^ of control cells (transfected with #Con1 & #Con2 SSOs) (Figure 6A). The cells transfected with Del2 & #Ins7 SSOs (expressing only the ∆2 splice variant) demonstrated slightly low proliferation capacity, and in 96 h, their numbers were not different from those of control cells (2.70 ± 0.28 × 10^7^). The use of SSO couples aimed at the deletion of exon 7 (#Ins2 & #Del7 and #Del2 & #Del7) significantly decreased the proliferation activity; 2.14 ± 0.13 × 10^7^ cells were obtained in Tregs expressing the ∆7 variant only, and 1.98 ± 0.09 × 10^7^ cells were obtained in Tregs expressing the ∆2∆7 variant only.

Transfection procedures and/or selective expression of a single FoxP3 splice variant may induce cell death. We monitored cell viability within 96 h after transfection by PI staining and observed significant induction of cell death only in 24 h (up to 10.12%) and in 48 h (up to 6.46%, Figure S10 in the Supplementary File). The percent of PI-positive cells in 72 or 96 h did not differ from non-transfected cells. This result demonstrated the toxicity of the transfection procedure but not each of the induced FoxP3 splice variants.

The rates of cell growth in the population and the divisions of individual cells may not coincide because the population of cells may contain a certain number of cells that are not capable of division. We labeled transfected Tregs with the vital dye CFSE and measured the percentage of proliferating cells by flow cytometry. The induction of the FL splice variant (transfection with #Ins2 and #Ins7) resulted in a significant increase in the proportion of proliferating cells, and at 96 h post-transfection, 98.04 ± 1.02% of cells exhibited rounds of division in comparison to 93.83 ± 2.02% of control cells (transfected with #Con1 and #Con2) (Figure 6B). In Figure S11 in the Supplementary File, we provided flow cytometry diagrams of proliferating CFSE-labeled Tregs transfected with SSOs. The induction of the ∆2 splice variant (transfection with #Del2 and #Ins7) led to the suppression of cell proliferation in 96 h, and 70.78 ± 3.87% of the cell population performed the divisions. The selective expression variants with deletions of exon 7 (∆7 and ∆2∆7 variants) were associated with a dramatic decrease in the proportion of proliferating cells: 41.14 ± 3.86% for #Ins2 and #Del7 transfection and 37.15 ± 4.34 for #Del2 and #Del7 transfection.

**Figure 7:**
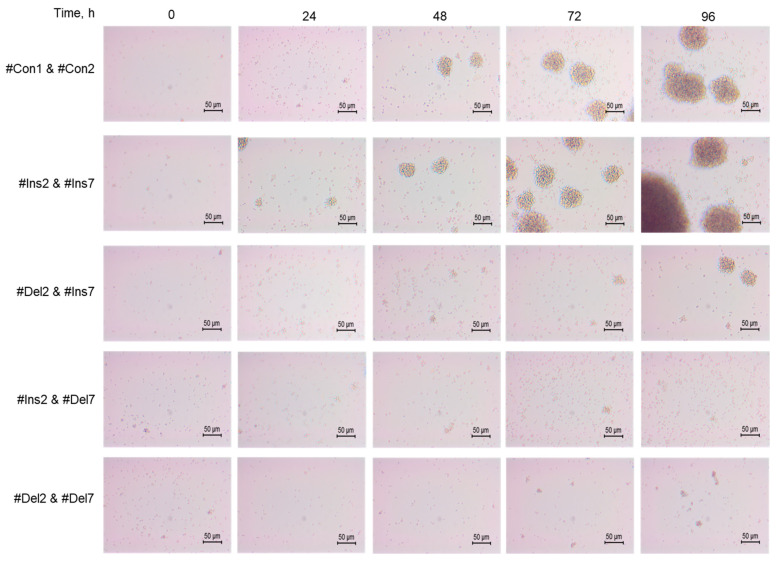
Development dynamics of Tregs transfected with oligonucleotides within 96 h of cultivation (see description in text). Microphotographs of proliferating cells at 200× magnification.

The morphology of cells was studied using an inverted microscope, and differences in the features of transfected cell proliferation were found. Control cells (#Con1 & #Con2) formed cell clusters or aggregates 60–80 μm in size (Figure 7). Tregs expressing the FL variant (#Ins2 & #Ins7 transfected) formed more aggregates with sizes up to 120 μm. The deletion of exon 2 (#Del2 & #Ins7 transfection) resulted in the formation of fewer aggregates with smaller sizes of approximately 50 μm and less. Tregs with deletions of exon 7 (transfected with #Ins2 & #Del7 or #Del2 & #Del7) were not able to form aggregates.

Measuring individual cell size by a cell analyzer demonstrated that cells lacking both exons 2 and 7 (transfected with #Del2 & #Del7 SSOs) had smaller diameters than those transfected with other nucleotides (Table 5), while the average circularity of individual cells did not change. In Figure S12 in the Supplementary File, we provided representative diagrams of the size distribution of individual transfected cells.

### 3.7. Expression of Treg-Associated Cell Markers in Cells Transfected with SSOs

A flow cytometry study was performed to analyze the expression of Treg-associated molecular markers in cells 96 h after transfection with the pairs of SSOs. In Figure S13 in the Supplementary File, we provided the results and representative flow cytometry diagrams demonstrating the proportion of cells with negative, low, and high expression of membrane surface cell markers.

**Figure 8:**
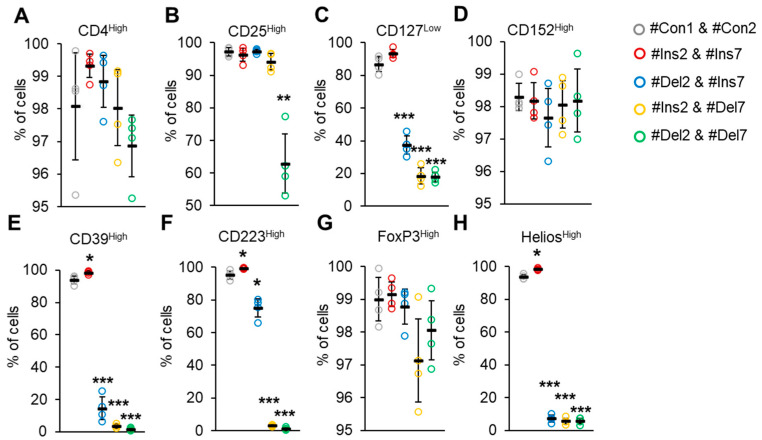
Immunophenotype of Tregs expressing only one FoxP3 splice variant. The expression of Treg-associated cell markers was determined by flow cytometry in Tregs four days after transfection with each of the SSOs. Cell membrane markers were (**A**) CD4^High^, (**B**) CD25^High^, (**C**) CD127^Low^, and (**D**) CD152^High^. Cell membrane markers associated with Treg suppressive activity were (**E**) CD39^High^ and (**F**) CD223^High^. Nuclear markers associated with Treg stability: (**G**) FoxP3^High^ and (**H**) Helios^High^. n = 4. Black horizontal lines indicate the mean ± SEM. * *p* ≤ 0.05; ** *p* ≤ 0.01; *** *p* ≤ 0.005 vs. cells transfected with #Con1 & #Con2 oligonucleotides by the Mann–Whitney U test.

The expression of the CD4 marker transmembrane immunoglobulin, which functions as a coreceptor for MHCII [34], was not different in all groups of transfected cells, and 96.9–98.1% of Tregs demonstrated the CD4^High^ phenotype (Figure 8A). The expression of the CD25 marker, the alpha subunit of the interleukin 2 receptor [35], was decreased only after transfection with #Del2 & #Del7: 62.9 ± 9.0% of cells had a CD25^High^ phenotype (Figure 8B). 

Peripheral Tregs are commonly characterized by low expression of CD127, an interleukin 7 receptor subunit alpha [36]. The CD127^Low^ phenotype was demonstrated only by cells transfected with #Con1 & #Con2 control and #Ins2 & #Ins7 SSOs (Figure 8C). Deletion of exon 2 (transfection with #Del2 & #Ins7) resulted in decreasing amounts of CD127^Low^ cells (37.3 ± 5.6%). The lowest amounts of cells exhibiting the CD127^Low^ phenotype were observed in cells with induced deletion of exon 7 (transfection with #Ins2 & #Del7) (18.5 ± 4.9%) and in cells with deletion of both exons (transfection with #Del2 & #Del7) (18.0 ± 2.9%). CD152 is cytotoxic T-lymphocyte-associated protein 4, an immunoglobulin capable of inhibiting immune reactions commonly exhibited by Tregs [37]. In our experiments, cells transfected with all types of SSOs demonstrated a CD152^High^ phenotype (Figure 8D) with no significant difference. CD39 is an ectonucleoside triphosphate diphosphohydrolase-1 that binds to adenosine receptor A2 on the surface of effector T lymphocytes and suppresses their proliferation by increasing cAMP levels [38]. We observed an increased proportion (98.2 ± 0.8%) of CD39^High^ cells after induction of the FL variant (transfection with #Ins2 & #Ins7, Figure 8E) in comparison to control cells (93.7 ± 2.4%). The induction of the ∆2 variant resulted in a decrease in such cells up to 10.7 ± 7.1%. The lowest proportions of CD39^High^ cells were observed after induction of the ∆7 variant (2.4 ± 1.3%) or ∆2∆7 variant (1.9 ± 0.7%). CD223 is a lymphocyte activation gene-3 that is responsible for binding with MHC-II molecules in antigen-presenting cells and triggering an inhibitory signaling pathway that prevents the activation of T lymphocytes [39]. We observed an increased proportion (99.6 ± 0.3%) of CD223^High^ cells after induction of the FL variant (Figure 8F) in comparison to control cells (96.0 ± 0.3%). The induction of the ∆2 variant resulted in a decrease in such cells of up to 78.4 ± 5.5%. The lowest proportions of CD223^High^ cells were observed after induction of the ∆7 variant (2.7 ± 0.5%) or ∆2∆7 variant (1.1 ± 0.7%). As described above, FoxP3 is a transcription factor, a master protein capable of regulating Treg proliferation, differentiation, and suppressive activity [7,8]. The use of commercially available antibodies did not allow us to detect Tregs expressing only one of four FoxP3 splice variants, and the proportions of FoxP3^High^ cells did not change after transfection with any of the SSO couples (Figure 8G). Another transcription factor involved in Treg stability is Helios, which belongs to the Ikaros family of zinc finger proteins [40]. An increased proportion of Helios^High^ cells (98.4 ± 0.5%) was observed after the induction of the FL variant (98.4 ± 0.5) in comparison to the control (93.7 ± 1.3%). Transfection of Tregs with any couple of SSOs led to a dramatic decrease in Helios-expressing cells: up to 7.2 ± 2.1% in cells with the induced ∆2 variant; 5.7 ± 1.8% in cells with the induced ∆7 variant; and 5.8 ± 1.8% in cells with the induced ∆2∆7 variant.

The levels of MFI for the studied cell markers are presented in Table S4 in the Supplementary File.

### 3.8. The Effect of FoxP3 Alternative Splicing on the Suppressive Activity of Tregs

**Figure 9:**
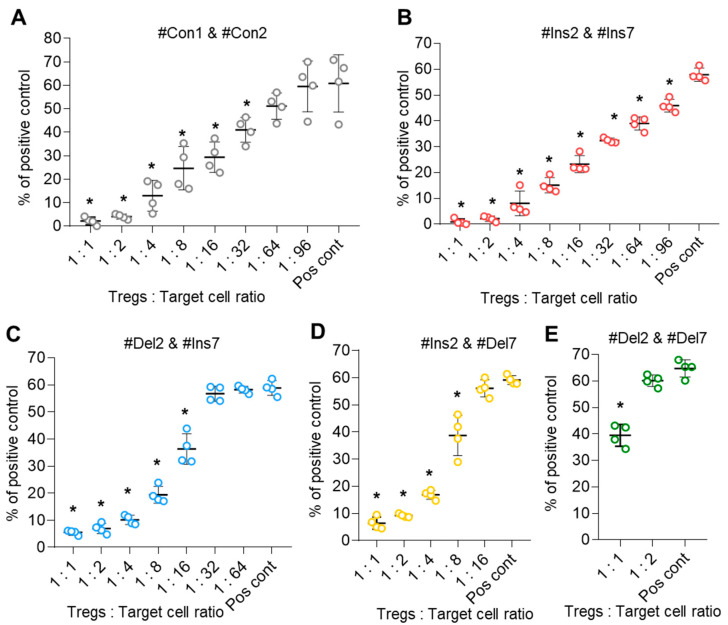
Tregs expressing only one FoxP3 splice variant demonstrated different suppressive activities. Tregs were transfected with each SSO, inducing the expression of only one FoxP3, and subjected to an MLR test at 96 h of proliferation. (**A**–**E**) The results of the MLR test demonstrate increased suppressive activity in cells expressing the FL variant and decreased suppressive activity in cells with exon deletions in splice variants. The results are shown as the percentage of proliferated cells measured by the reduction in the CFSE signal using flow cytometry. Representative flow cytometry diagrams for the MLR test are shown in Figure S14 in the Supplementary File. Pos. cont.—positive control; CD4^+^CD25^−^ cells cocultured with accessory cells. n = 4 for both the MS and HD groups. * *p* ≤ 0.05 by the Student’s *t* test.

The suppressive activity of transfected Tregs expressing only one FoxP3 splice variant was studied in an MLR assay. Control cells (transfected with #Con1 & #Con2 oligonucleotides) suppressed the proliferation of target cells at a ratio of 1:32 (Figure 9A). The suppressive activity of Tregs expressing the FL variant (transfection with #Ins2 & #Ins7) was three times higher, and they could suppress target cells at a ratio of 1:96 (Figure 9B). Diminished suppressive activity was observed in Tregs with FoxP3 splice variants with exon deletions. Tregs with the ∆2 variant (transfection with #Del2 & #Ins7) demonstrated suppressive activity at a ratio of 1:16 (Figure 9C), and Tregs with the ∆7 variant (transfection with #Ins2 & #Del7) demonstrated suppressive activity at a ratio of 1:8 (Figure 9D). The weakest suppressive activity was observed in Tregs expressing the ∆2∆7 variant (transfected with #Del2 & #Del7). Such cells were active only at a ratio of 1:1 (Figure 9E). Therefore, the results of this experiment demonstrated that Tregs with truncated FoxP3 have decreased suppressive activity.

### 3.9. The Effect of FoxP3 Alternative Splicing on Treg-Associated Suppressive Molecules

**Figure 10:**
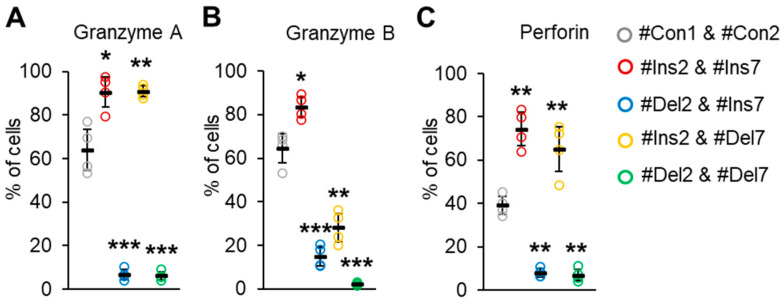
The ability of Tregs to produce apoptosis-inducing molecules (**A**) granzyme A, (**B**) granzyme B, and (**C**) perforin. Transfected Tregs were incubated with stimulators and protein transport inhibitors or only with protein transport inhibitors as a control. Cells were labeled with CD4, CD25, and CD127 antibodies, fixed, permeabilized, and incubated with antibodies against granzyme A, B, or perforin. Levels of marker-positive cells were measured by flow cytometry. n = 4. Black horizontal lines indicate the mean ± SD. * *p* ≤ 0.05; ** *p* ≤ 0.01; *** *p* ≤ 0.005 vs. cells transfected with #Con1 & #Con2 oligonucleotides by the Mann–Whitney U test. Representative flow cytometry diagrams for granzymes A and B and perforin are shown in Figure S15 in the Supplementary File.

We examined the ability of transfected Tregs to synthesize molecules involved in suppressive activity. The serine proteases granzyme A, granzyme B, and granzyme-associated perforin are responsible for contact-dependent suppressive activity [41]. We observed a decrease in cells producing these molecules upon deletion of exon 2, but not exon 7. Almost all the cells with the FL variant (transfected with #Ins2 & #Ins7, 90.6 ± 6.9%) or ∆7 variant (transfected with #Ins2 & #Del7, 90.9 ± 2.5%) were granzyme A-positive (Figure 10A) in comparison to control cells (transfected with #Con1 & #Con2, 64.9 ± 9.6%).

Tregs expressing variants with deleted exon 2, i.e., transfected with #Del2 & #Ins7 or #Del2 & #Del7, demonstrated a granzyme A-negative phenotype (6.7 ± 2.2% and 6.2 ± 1.9%, respectively). The proportion of granzyme B-positive cells was also increased with the induction of the FL variant (83.5 ± 5.1%) in comparison to control cells (64.5 ± 7.2%) (Figure 10B). The deletion of exon 7 was associated with a slight decrease in cells with granzyme B (38.3 ± 6.9%). Tregs with the deletion of exon 2 (∆2 or ∆2∆7 variants) demonstrated the lowest rates of granzyme B synthesis (15.1 ± 4.6% and 2.5 ± 0.6%, respectively). The proportion of perforin-positive control cells was 39.2 ± 4.8% and increased up to 74.4 ± 8.9% after the induction of the FL variant or up to 65.1 ± 12.1% after the induction of the ∆7 variant (Figure 10C). Tregs with induced ∆2 or ∆2∆7 variants had a diminished capacity for perforin synthesis (7.9 ± 2.1% and 7.0 ± 3.1%, respectively).

**Figure 11:**
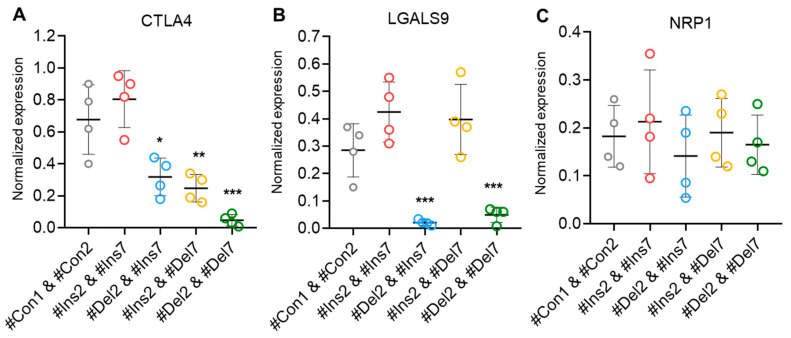
Levels of mRNA of molecules associated with Treg suppressive activity. Total mRNA was isolated from Tregs transfected with oligonucleotides and analyzed by real-time RT-PCR. The mRNA levels of (**A**) CTLA4, (**B**) LGALS9, and (**C**) NRP1 were normalized to the mean expression of three reference genes: 18S, GAPDH, and ACTB. n = 4. * *p* ≤ 0.05; ** *p* ≤ 0.01; *** *p* ≤ 0.005 vs. cells transfected with #Con1 & #Con2 oligonucleotides by the Mann–Whitney U test.

Treg contact-dependent suppression is also achieved via the expression of cytotoxic T-lymphocyte-associated protein 4 (CTLA4), which binds to costimulatory CD80/86 molecules on dendritic cells and enables them to activate target T cells [42]. Using real-time RT-PCR, we observed a decrease in the mRNA of this protein in Tregs with exon deletions (Figure 11A). In cells with the FL FoxP3 variant, the mRNA level did not differ from control cells, while in cells with ∆2 or ∆7 variants, its level was two times lower. This observation is not in accordance with the results of the CD152 analysis by flow cytometry (Figure 8D). The possible explanation is related to the previous observation that the FoxP3 truncated variant is less efficient at either inducing or maintaining expression of Treg-associated genes but is sufficient to generate a suppressive Treg phenotype [12]. The cells with the ∆2∆7 variant had a tenfold lower rate of mRNA expression. Galectin 9 (LGALS9) is a family of beta-galactoside-binding proteins implicated in modulating cell-cell interactions and is involved in Treg suppressive function by binding to TNF receptor superfamily member 25 in cytotoxic cells [43]. Only cells with induced ∆2 and ∆2∆7 variants demonstrated downregulated levels of this protein (Figure 11B). Neither the induction of the ∆7 variant nor the FL variant resulted in changes in LGALS9 mRNA levels. Neuropilin-1 (NRP1) is required for Treg-mediated immunosuppression via its impact on the phenotype and function of effector T lymphocytes [44]. We observed no difference in the mRNA levels of this protein among transfected Tregs (Figure 11C).

**Figure 12:**
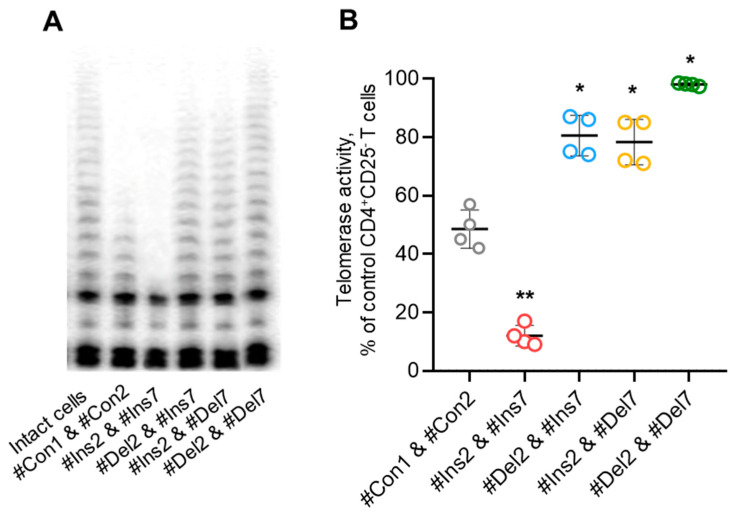
Tregs expressing only one FoxP3 splice variant demonstrated different abilities to suppress telomerase. (**A**) Telomerase activity determined by TRAP assay. (**B**) Results of TRAP quantification by densitometry. * *p* ≤ 0.05; ** *p* ≤ 0.01 vs. cells transfected with #Con1 & #Con2 oligonucleotides by the Mann–Whitney U test.

Tregs can perform suppressive activity in a contact-independent manner via downregulation of telomerase in target cells [27]. Using the TRAP assay, we demonstrated that Tregs with FL variants could inhibit telomerase significantly more effectively than those with exon 2 and/or 7 deletion variants (Figure 12). Moreover, the cells with deletion variants suppressed telomerase less effectively than control cells. Thus, the results of these experiments demonstrated that Tregs with induced expression of FoxP3 splice variants have a diverse spectrum of molecules that are associated with suppressive activity.

### 3.10. Induction of FoxP3 Alternative Splicing Changes the Cytokine Profile of Tregs

**Figure 13:**
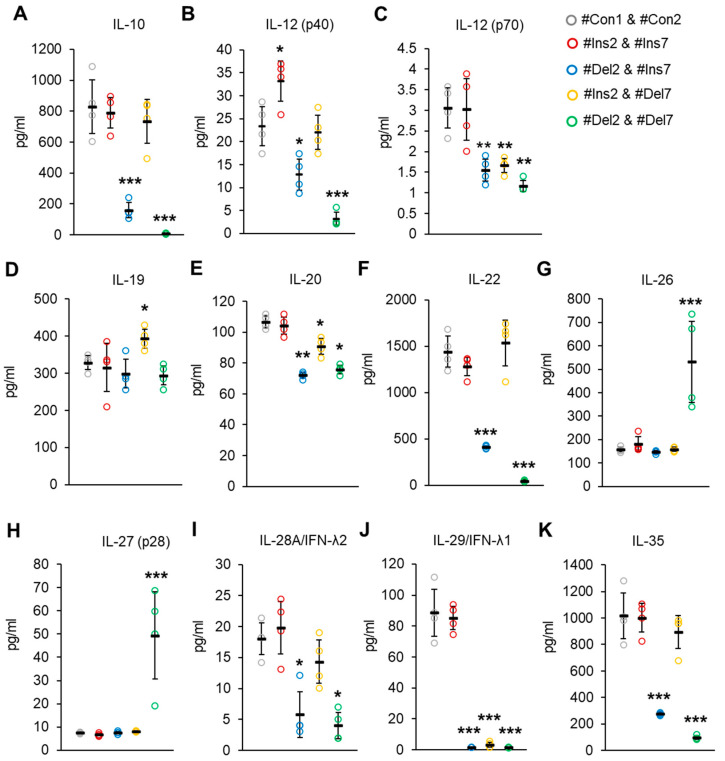
Treg-associated cytokine concentrations in cell culture from Tregs expressing a single FoxP3 splice variant. Concentrations of (**A**) IL-10, (**B**) IL-12 (p40), (**C**) IL-12 (p70), (**D**) IL-19, (**E**) IL-20, (**F**) IL-22, (**G**) IL-26, (**H**) IL-27 (p28), (**I**) IL-28A/IFN-λ2, (**J**) IL-29/IFN-λ1, and (**K**) IL-35 were determined by Bio-Plex assay. n = 4. Black horizontal lines indicate the mean ± SD. * *p* ≤ 0.05; ** *p* ≤ 0.01; *** *p* ≤ 0.005 vs. cells transfected with #Con1 & #Con2 oligonucleotides by the Mann–Whitney U test.

The concentrations of Treg-associated cytokines were measured by a Bio-Plex assay in cell culture media from transfected Tregs. The synthesis of cytokines involved in the suppressive activity of IL-10 and IL-35 [45,46] was suppressed only in cells with deletions of exon 2 (i.e., #Del2 & #Ins7- or #Del2 & #Del7-transfected cells, Figure 13A,K). Heterodimer IL-12 (p70) has the subunit p40 [47], which is responsible for enhancing the activation and proliferation of Tregs [48]. In our experiment, the concentrations of IL-12 (p40) were significantly higher in the media from cells with the FL variant (#Ins2 & #Ins7 transfected cells) and decreased in cells with the deletion of exon 2 (Figure 13B). Bioactive IL-12 (p70) was decreased in cells with any truncated FoxP3 splice variant (Figure 13C). IL-19, IL-20, IL-22, IL-28, and IL-29 belong to a family of negative-feedback regulators that limit the proinflammatory response [49]. IL-19 was slightly elevated only in cells with the deletion of exon 7 (#Ins2 & #Del7 transfected cells, Figure 13D). The IL-20 concentration was decreased in cells with any truncated variant (Figure 13E), and the concentrations of IL-22 were decreased only in cells transfected with #Del2 & #Ins7 or #Del2 & #Del7 but not those transfected with #Ins2 & #Del7 (Figure 13F). The concentrations of IL-28 decreased only in cells with deletions of exon 2 (Figure 13I), while the IL-29 concentration was decreased in cells with any truncated variant (Figure 13J). IL-26, which has proinflammatory activity, was significantly elevated only in #Del2- and #Del7-transfected cells (Figure 13G), which supports the observation that Tregs do not express IL-26 [50,51]. The same observation was made for IL-27 (p28) (Figure 13H), which can enhance the immune response [52].

Thus, the results of these experiments demonstrated that Tregs expressing ∆2, ∆7, or ∆2∆7 FoxP3 splice variants produce cytokines involved in cell development or suppressive activity less efficiently than cells with the FL variant, which corresponds to the affected suppressive activity of cells with truncated variants.

## 4. Discussion

Imbalances in Treg development and function have been observed during the setting and progression of different pathological disorders. The main finding of this study is the association of FoxP3 splice variants with the Treg phenotype and their proliferative and suppressive activity. While high and stable FoxP3 expression is considered the main factor for maintaining the stability of these cells [7,53], the involvement of its splice variants also has an effect on Treg biology in human and in vitro experiments. Tregs expressing truncated splice variants demonstrated a lower proliferation rate and suppressive activity than those expressing FL variants. Reduced suppression was associated with the decreased production of Treg-associated suppressive surface molecules and cytokines.

Most studies focus on Treg subsets expressing levels of individual cell markers that are believed to be associated with a disorder. A bright example is autoimmunity, in which abnormal Treg function or numbers are associated with the transcription factors GATA3 [54] and Tbet [55], the chemokines CXCR3 [56] and CXCR5 [57], and the cytokines interferon gamma [58], interleukin 5 [59], interleukin 4 [60], and interleukin 17 [61]. Interpretation of data based on the identification of cellular markers are now very variable and sometimes rather contradictory [62,63] because the molecular or genetic reasons resulting in the changed Treg phenotype are still open to study.

In our work, we demonstrated for the first time that peripheral blood Tregs from patients with diagnosed MS have different profiles of the expression of FoxP3 splice variants in comparison to HD (Figure 1). An important observation following this experiment is that all FoxP3 truncated variants (i.e., ∆2, ∆7, and ∆2∆7) exist in HDs, with an increased proportion in MS patients. The predominant expression of ∆2 and/or ∆7 variants in MS patients was in accordance with the reduced number of Tregs in peripheral blood that was previously described in many studies [64,65,66,67,68]. We also observed decreased total FoxP3 mRNA in Tregs from MS patients, which was also shown previously [69,70]. These data are in good agreement with those from another study demonstrating the development of immunodeficiency, polyendocrinopathy, and enteropathy X-linked (IPEX) syndrome in patients expressing only the shorter variant [12].

In our work, there were no correlations between FoxP3 splice variants and the clinical characteristics of the patient population (disease duration, MS course, predominant functional system involvement). 

Given that all MS patients included in this study had various clinical phenotypes of the disease and different disease courses (RRMS or SPMS), as well as highly variable disease duration ranging from 2 months to 27.5 years, this could indicate immunopathological heterogeneity of the patient population as well. 

There was also no statistically significant difference between the subgroup of patients with highly active MS and the rest of the MS group. This could be due to the fact that although the diagnosis of highly active MS implies high relapse frequency and high radiological activity, as well as accelerated accrual of disability in MS, it is not a specific disease subtype but solely a measure of MS activity, so patients with highly active MS can encompass a spectrum of diverse clinical and immunological phenotypes.

The protocol for ex vivo cultivation [19] allowed us to obtain Tregs suitable for functional tests and for transfection. The number of cells expressing CD127^low^ and FoxP3 protein (Figure 2) was determined in expanded Tregs to demonstrate the homogeneity of the population of obtained cells. Three main findings followed from this experiment. First, Tregs from MS patients have decreased proliferative speed, which is in agreement with reduced Treg quantities in the peripheral blood of MS patients [64,65,66,67,68]. Second, the suppressive activity of expanded Tregs is also reduced in comparison to that of HDs [3]. Third, the proportion of FoxP3 splice variants is retained after expansion in both MS and HDs. Thus, we concluded that ex vivo expansion had no dramatic impact on FoxP3 AS and the suppressive activity of Tregs, and we used expanded cells for further studies.

Modulation of pre-mRNA AS by SSO targeting splicing regulatory *cis*-elements has been shown by us in several studies on other objects [21,33,71], and we used this approach to induce a single FoxP3 splice variant in Tregs. As expected, the use of a single SSO inducing only one exon (2 or 7, Figure 3 and Figure 4) insertion or deletion did not allow us to obtain cells with only one splice variant. This is explained by the origination of all four FoxP3 splice variants from a single pre-mRNA, and the modulation of one exon has no effect on another. The mRNA rate of variants with nonmodulated exons remained unchanged. The use of couples of SSOs targeting both exons simultaneously allowed us to obtain cells expressing only one of the FoxP3 splice variants.

According to available information, the deletion of exon 2 results in the loss of a protein with an N-terminal proline-rich domain [72,73] responsible for the interaction with retinoic acid-related orphan receptors ROR alpha and ROR gammat and their inhibition that prevents the development of Th17 cells [74,75]. The deletion of exon 8 leads to the loss of the leucine zipper domain responsible for homo-oligomerization and heteroassociation with another transcription factor, FoxP1 [76,77]. However, Th17 differentiation can be stimulated by the ∆2∆7 splice variant [78]. We believe that different spectra of FoxP3 splice variants define ways to control the involvement of Tregs in immunoregulation at various levels. The next experiments of this study were devoted to characterizing the phenotype and functional activity of such Tregs and partially confirmed this hypothesis. Tregs expressing a single truncated splice variant had a reduced proliferation rate (Table 4, Figure 6), a changed immunophenotype (Figure 8), and decreased suppressive activity (Figure 8).

Human Tregs are most commonly defined as cells with a CD4^+^CD25^+^CD127^low^Foxp3^+^ phenotype; however, the use of Treg markers is not consistent. Several studies have demonstrated that some MS patients have no detectable CD127^low^ Tregs [29]. According to our data (Figure 8), we propose that the induction of FoxP3 splice variants in Tregs of such patients may be the reason for this observation.

In our study, the molecules involved in suppressive activity were monitored. We observed the downregulation of CD39 (Figure 8E), CD223 (Figure 8F), granzymes A and B, perforin (Figure 10), CTLA 4, and LGALS9 (Figure 11) upon the induction of truncated splice variants.

The decreased CD39 and perforin levels are in contrast with other data demonstrating an increase in CD39^+^ Tregs [79,80] and perforin expression in lymphocytes from MS patients [81]. However, the decrease in LAG-3 (CD223) T cells in MS has been confirmed in several studies [82,83].

The impaired suppressive and proliferative activity of Tregs expressing a single truncated variant is in good agreement with their reduced ability to synthesize cytokines (Figure 13), which are capable of stimulating cell growth and proliferation (IL-12), inhibitory activity (IL-10, IL-35), and anti-inflammatory activity (IL-19, IL-20, IL-22, IL-28, and IL-29). 

Although the expression of FoxP3 is considered the definitive marker of Tregs, several studies have suggested that it can also be expressed in human activated nonregulatory CD4^+^ T cells [84,85,86]. In addition to the roles of Tregs in maintaining immune homeostasis in lymphoid tissues, Treg cells reside in healthy and tumor tissues [87]. Such heterogeneity in FoxP3-positive cells is associated with their phenotypic dynamics. Induction of individual FoxP3 splice variants in various cells may become a key to understanding the development of an associated cellular phenotype and function.

Several limitations must be overcome for better investigation of FoxP3 AS in Tregs in future works.

First, this study was performed without controlling the protein levels of FoxP3 splice variants, which raises the issue of obtaining antibodies that are capable of detecting each single splice variant in cells. Antibodies capable of detecting FL and the ∆3 variant were used in a recent study [14], but antibodies for the ∆8 and ∆3∆8 variants remain to be produced. However, real-time RT-PCR with the provided primers could successfully detect the proportion of FoxP3 splice variants in our experiments.

Second, the results of this work also indicate the heterogeneity of Tregs, even in individual MS patients and HDs. Thus, the analysis of all isolated control cells gives an average level of FoxP3 splice variants and cellular markers. We believe that single-cell proteomic analysis can provide a stronger association between FoxP3 AS and Treg-associated molecules.

Third, very few Treg cells could be isolated from peripheral blood, which makes functional tests difficult. Although we demonstrated that ex vivo multiplication of cells does not lead to changes in the profile of FoxP3 splice variants, this procedure could affect other cellular parameters, making them different from those in peripheral blood. Partially, this issue can be resolved by the abovementioned single-cell proteomic study, but functional tests with small quantities of cells need to be elaborated.

Fourth, modulation of AS by splice-switching oligonucleotides provides reliable but temporary induction of selective expression of FoxP3 splice variants. Gene editing using the CRISPR/Cas technique for exon deletion or editing nucleotides in splicing regulatory regions can help to obtain stable Treg cell lines with stable expression of a single splice variant.

Nevertheless, in this work, we demonstrated that modulation of FoxP3 alternative splicing toward the induction of FL variants induces the functional suppressive activity of Tregs. We can speculate that, in the case of further development, this approach can become a new strategy for regenerative support of autoimmune disease treatment.

## Figures and Tables

**Table 1:** Splice-switching oligonucleotides are used for Treg transfection.

SSO	Sequence (5′–3′)	Label
#Con1	A*T*G*T*G*CCGTAGGTGAGGCCTCACGTTCGTTA*A*A*C*G*G	Cy5.5
#Con2	G*T*G*A*G*GCCTCACGTTCGTTAAACGGATGTGC*C*G*T*A*G	Cy3
#Ins2	A*T*G*T*G*GCCTGTCCAGGAGGAGTGCCTGTAAG*T*G*G*G*G	Cy5.5
#Ins7	G*G*C*A*C*TCACGTTCTCCTTCTCCAGCACCAGC*T*G*T*G*A	Cy3
#Del2	T*C*C*C*A*CCTGCTCCCTCCTCCCTGCCCATTCA*C*C*G*T*C	Cy5.5
#Del7	C*T*G*C*A*CCGTGCAGACCTCCTCCCTGCCCCCC*A*G*C*A*G	Cy3

*—positions of 2′MOE-modified oligonucleotides.

**Table 2:** Demographics of MS patients and healthy donors.

Parameter	MS Group (n = 20)	HD Group (n = 20)
Number of female patients (%)	15 (75%)	12 (62.8%)
Age at study enrollment, mean ± SD *	37.1 ± 11.1	36.2 ± 12.2
Age range	20–59	21–58

* SD—standard deviation (95% confidence interval).

**Table 3:** Clinical data of MS patients. All data are presented as the mean ± SD, with a 95% confidence interval.

Parameter	MS Group (n = 20)
Disease duration (years)	6.1 ± 7.3 (0.1–27.5)
EDSS	3.5 ± 1.3 (1.5–6)
MS course, patients (%)	
relapsing-remitting	15 (75%)
active, secondary, and progressive	3 (15%)
non-active, secondary, or progressive	2 (10%)
Patients experiencing an MS relapse at study enrollment (%)	10 (50%)
Functional system involvement, patients (%)	
pyramidal	18 (90%)
cerebellar	18 (90%)
brainstem	18 (90%)
sensory	17 (85%)
bowel/bladder	9 (45%)
visual	8 (40%)
mental	10 (50%)
ambulation	12 (60%)
Highly active MS patients (%)	6 (30%)
DMT-naïve patients (%)	15 (75%)

**Table 4:** Frequency of cell divisions per day in Tregs transfected with SSOs and selectively expressing a single FoxP3 splice variant.

SSOs Transfection	Cell Divisions per Day
#Con1 & #Con2	0.40
#Ins2 & #Ins7	0.58
#Del2 & #Ins7	0.36
#Ins2 & #Del7	0.27
#Del2 & #Del7	0.25

**Table 5:** Average diameter and circularity of Tregs transfected with SSO 96 h after transfection.

SSOs Transfection	Average Diameter (Microns)	Average Circularity
#Con1 & #Con2	10.65	0.89
#Ins2 & #Ins7	10.64	0.88
#Del2 & #Ins7	10.84	0.86
#Ins2 & #Del7	10.24	0.85
#Del2 & #Del7	7.76	0.86

## Data Availability

Data are available upon request via the corresponding author.

## Supplementary Materials

The following supporting information can be downloaded at: https://www.mdpi.com/article/10.3390/cells13010077/s1. Figure S1: Gating strategy for Treg determination in peripheral blood from MS patients or HDs; Figure S2: FoxP3 splice variant mRNA expression in Tregs isolated from the peripheral blood of MS patients and HDs; Figure S3: Gating strategy demonstrating the purity of isolated Tregs from MS patients and HDs; Figure S4: Gating strategy demonstrating the homogeneity of ex vivo multiplicated Tregs from MS patients and HDs for 15 days; Figure S5: Representative proliferative diagrams of the mixed lymphocyte reaction study for ex vivo expanded Tregs isolated from MS patients and HDs; Figure S6: Transfection efficiency of Tregs with a single SSO targeting exon 2 or exon 7; Figure S7: FoxP3 splice variant mRNA expression in nontransfected Tregs and Tregs transfected with the control nonspecific oligonucleotide #Con1 96 h after transfection; Figure S8: Transfection efficiency of Tregs with the couple SSOs targeting both exon 2 and exon 7; Figure S9: Total FoxP3 expression in Tregs transfected with SSO or control oligonucleotides; Figure S10: Monitoring of cell death by flow cytometry using Propidium Iodide (PI) staining; Figure S11: Proliferation of Tregs transfected with SSO; Figure S12: Size distribution of Tregs transfected with SSOs; Figure S13: Flow cytometry study of Treg-associated cell markers; Figure S14: Representative proliferative diagrams of the mixed lymphocyte reaction study for transfected Tregs: Figure S15: The ability of Tregs to produce apoptosis-inducing molecules: granzyme A, granzyme B, and perforin; Table S1: List of primers used for real-time RT–PCR; Table S2: Proportion of Tregs in peripheral blood and the expression of FoxP3 and FoxP3 splice variants in Tregs isolated from peripheral blood from multiple sclerosis patients; Table S3: Expression of FoxP3 and FoxP3 splice variants in Tregs isolated from peripheral blood from healthy donors; Table S4: Mean fluorescent intensities of Treg-associated cell markers.
