# Supplementary material for: Modulation of Suppressive Activity and Proliferation of Human Regulatory T Cells by Splice-Switching Oligonucleotides Targeting FoxP3 Pre-mRNA
Source: Cells. 2023 Dec 29;13(1):77. doi: 10.3390/cells13010077 (PMC10777989; doi:10.3390/cells13010077)
Supplement: Supplementary file 1 [file cells-13-00077-s001.zip › cells-2738424-supplementary.pdf]

Modulation of suppressive activity and proliferation of human regulatory T cells by splice-switching oligonucleotides targeting FoxP3 pre-mRNA

Varvara G. Blinova[1], Yulia A. Gladilina[1], Anna A. Abramova[2], Daria D. Eliseeva[2], Valentina V. Vtorushina[3], Anastasia N. Shishparenok[1], and Dmitry D. Zhdanov[1,4,*]

[1] Institute of Biomedical Chemistry, Laboratory of Medical Biotechnology, Pogodinskaya st. 10/8, 119121, Moscow, Russia.

[2] Research Center of Neurology, Volokolamskoe shosse, 80, 125367, Moscow, Russia.

[3] National Medical Research Center for Obstetrics, Gynecology and Perinatology named after Academician V.I. Kulakov of Ministry of Healthcare of Russian Federation, Laboratory of Clinical Immunology, Academician Oparin st. 4., 117997, Moscow, Russia.

[4] Patrice Lumumba People's Friendship University of Russia (RUDN University), Department of biochemistry, Miklukho-Maklaya st. 6, 117198, Moscow, Russia.

* Corresponding author at: Institute of Biomedical Chemistry, Pogodinskaya st 10/8, Moscow, 119121, Russia.
Email address: zhdanovdd@gmail.com (D.D. Zhdanov).

Content

Figure S1. Gating strategy for Treg determination in peripheral blood from MS patients or HDs. 3

Table S1. List of primers used for real-time RT–PCR. 4

Figure S2. FoxP3 splice variant mRNA expression in Tregs isolated from the peripheral blood of MS patients and HDs. 5

Table S2. Proportion of Tregs in peripheral blood and the expression of FoxP3 and FoxP3 splice variants in Tregs isolated from peripheral blood from multiple sclerosis patients. 6

Table S3. Expression of FoxP3 and FoxP3 splice variants in Tregs isolated from peripheral blood from healthy donors. 7

Figure S3. Gating strategy demonstrating the purity of isolated Tregs from MS patients and HDs. 8

Figure S4. Gating strategy demonstrating the homogeneity of *ex vivo* multiplicated Tregs from MS patients and HDs for 15 days. 9

Figure S5. Representative proliferative diagrams of the mixed lymphocyte reaction study for *ex vivo* expanded Tregs isolated from MS patients and HDs. 10

Figure S6. Transfection efficiency of Tregs with a single SSO targeting exon 2 or exon 7. 11

Figure S7. FoxP3 splice variant mRNA expression in nontransfected Tregs and Tregs transfected with the control nonspecific oligonucleotide #Con1 96 h after transfection. 12

Figure S8. Transfection efficiency of Tregs with the couple SSOs targeting both exon 2 and exon 7. 13

Figure S9. Total FoxP3 expression in Tregs transfected with SSO or control oligonucleotides. 14

Figure S10. Monitoring of cell death by flow cytometry using Propidium Iodide (PI) staining. 15

Figure S11. Proliferation of Tregs transfected with SSO. 16

Figure S12. Size distribution of Tregs transfected with SSOs. 17

Figure S13. Flow cytometry study of Treg-associated cell markers. 18

Table S4. Mean fluorescent intensities of Treg-associated cell markers. 19

Figure S14. Representative proliferative diagrams of the mixed lymphocyte reaction study for transfected Tregs. 20

Figure S15. The ability of Tregs to produce apoptosis-inducing molecules: granzyme A, granzyme B, and perforin. 21

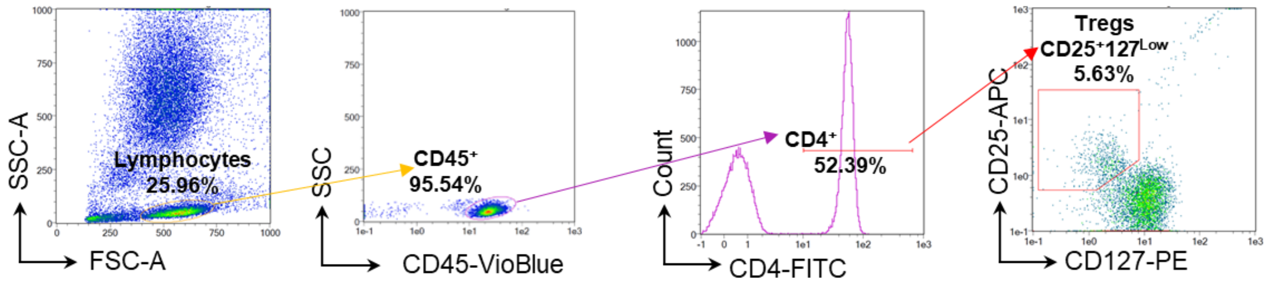

Figure S1. Gating strategy for Treg determination in peripheral blood from MS patients or HDs. Representative flow cytometry diagrams of 40 (20 MS patients and 20 healthy donors) blood samples are shown. The study was performed by cell labeling with antibodies from Treg Surface Marker Analysis Cocktail, anti-human (130-096-082, Miltenyi Biotec, Miltenyi Biotec, Bergisch Gladbach, Germany) using flow cytometer MACSQuant Analyzer 10 (Miltenyi Biotec, Bergisch Gladbach, Germany).

Table S1. List of primers used for real-time RT–PCR.

Target	Sense 5’ – 3’	Antisense 5’ – 3’	Product size, bp.	Tann, °C
FoxP3 total	agagagcctgcctcagtaca	tgacgctgcttctgtgtagg	178	60
FL FoxP3	ctagtcatggtggcaccctc	gatgaagccttggtcagtgcc	587	59
Δ2 FoxP3	cagctgcagctctcaacggtg	gatgaagccttggtcagtgcc	506	61
Δ7 FoxP3	ctagtcatggtggcaccctc	gtcggatgatgcctgctgctc	522	60
Δ2Δ7 FoxP3	cagctgcagctctcaacggtg	gtcggatgatgcctgctgctc	441	62
CTLA4	aggtgactgaagtctgtgcg	aagtcagaatctgggcacgg	247	59
LGALS9	tccagctgtccccttttctg	agcagaggtcaaagggcatc	272	60
NRP1	ctccacgcgattcatcagga	ccacctgtgagctggaagtc	198	60
18S	ggatccattggagggcaagt	acgagctttttaactgcagcaa	91	62
Beta-actin	cctgggcatggagtcctgt	atctccttctgcatcctgtcg	153	60
GAPDH	gaaggtgaaggtcggagtc	gaagatggtgatgggatttc	226	57

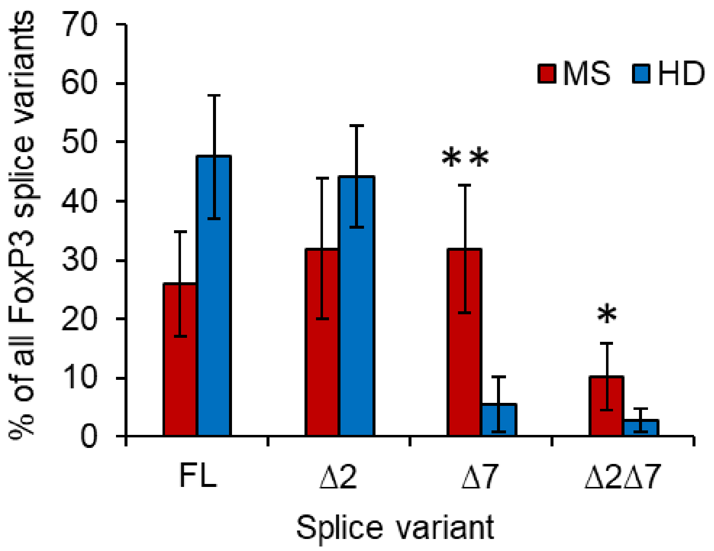

Figure S2. FoxP3 splice variant mRNA expression in Tregs isolated from the peripheral blood of (A) MS patients and (B) HDs. The levels of mRNA were determined by real-time RT–PCR as described in the Materials and Methods. N=20 for each MS and HD group. The percentage of each splice variant is shown. All FoxP3 splice variants. $^*p \leq 0.05$; $^{**}p \leq 0.01$ by Mann–Whitney U test. FL, full-length variant; Δ2, variant with the deletion of exon 2; Δ7, variant with the deletion of exon 7; Δ2Δ7, variant with the deletion of both exons 2 and 7.

Table S2. Proportion of Tregs in peripheral blood and the expression of FoxP3 and FoxP3 splice variants in Tregs isolated from peripheral blood from multiple sclerosis patients.

MS patient	Treg, % of CD4$^+$	Total FoxP3	FL variant, % of total splice variants	Δ2 variant, % of total splice variants	Δ7 variant, % of total splice variants	Δ2Δ7 variant, % of total splice variants
1	3.69	0.291	39.1	21.1	33.5	6.4
2	3.87	0.295	38.9	21.8	26.5	12.8
3	4.67	0.286	35.0	26.2	30.0	8.9
4	4.16	0.258	34.7	32.9	29.7	2.7
5	4.21	0.205	33.7	25.3	37.3	3.6
6	2.56	0.380	32.8	16.3	40.6	10.3
7	5.15	0.240	32.5	33.5	26.7	7.3
8	5.3	0.278	30.7	36.0	24.3	9.0
9	6.36	0.274	28.7	33.2	30.0	8.1
10	3.81	0.386	28.0	36.0	22.1	13.9
11	6.85	0.377	25.9	43.1	17.9	13.1
12	8.07	0.308	25.7	24.3	44.6	5.4
13	5.90	0.204	23.0	49.4	18.0	9.6
14	5.15	0.350	20.9	19.0	32.1	28.0
15	6.23	0.201	20.5	63.9	12.7	3.0
16	7.24	0.305	18.2	28.1	42.3	11.3
17	5.51	0.163	16.8	27.7	37.4	18.1
18	2.12	0.188	13.3	17.6	60.0	9.1
19	3.98	0.213	11.0	42.1	35.9	11.0
20	2.06	0.269	6.9	16.7	66.7	9.7

FL, full-length variant; MS, multiple sclerosis; Δ indicates the number of deleted exons.

Table S3. Expression of FoxP3 and FoxP3 splice variants in Tregs isolated from peripheral blood from healthy donors.

HD	Treg, % of CD4$^+$	Total FoxP3	FL variant, % of total splice variants	Δ2 variant, % of total splice variants	Δ7 variant, % of total splice variants	Δ2Δ7 variant, % of total splice variants
1	9.55	0.626	75.0	23.1	1.1	0.9
2	5.8	0.473	67.0	25.4	6.2	1.3
3	6.3	0.751	63.4	32.5	3.2	0.9
4	7.7	0.638	53.7	42.4	3.0	0.8
5	11.54	0.475	51.5	44.5	1.8	2.2
6	8.01	0.722	51.4	46.5	1.1	1.1
7	6.81	0.601	47.4	37.9	12.6	2.2
8	8.85	0.654	47.4	48.3	2.7	1.6
9	10.04	0.411	46.0	50.6	1.1	2.3
10	5.63	0.451	45.5	47.6	3.9	2.9
11	8.47	0.49	42.9	54.2	1.2	1.8
12	8.12	0.42	42.4	50.0	5.3	2.3
13	8.21	0.697	42.4	47.5	4.4	5.8
14	9.83	0.388	42.3	54.3	0.9	2.6
15	11.06	0.45	41.5	44.5	11.8	2.1
16	10.2	0.801	40.6	40.3	13.4	5.7
17	8.28	0.435	39.9	54.0	1.9	4.1
18	8.0	0.636	38.4	44.5	10.0	7.1
19	8.85	0.399	36.8	47.0	10.3	5.9
20	8.07	0.415	35.4	48.6	13.4	2.6

FL, full-length variant; HD, healthy donors; Δ indicates the number of deleted exons.

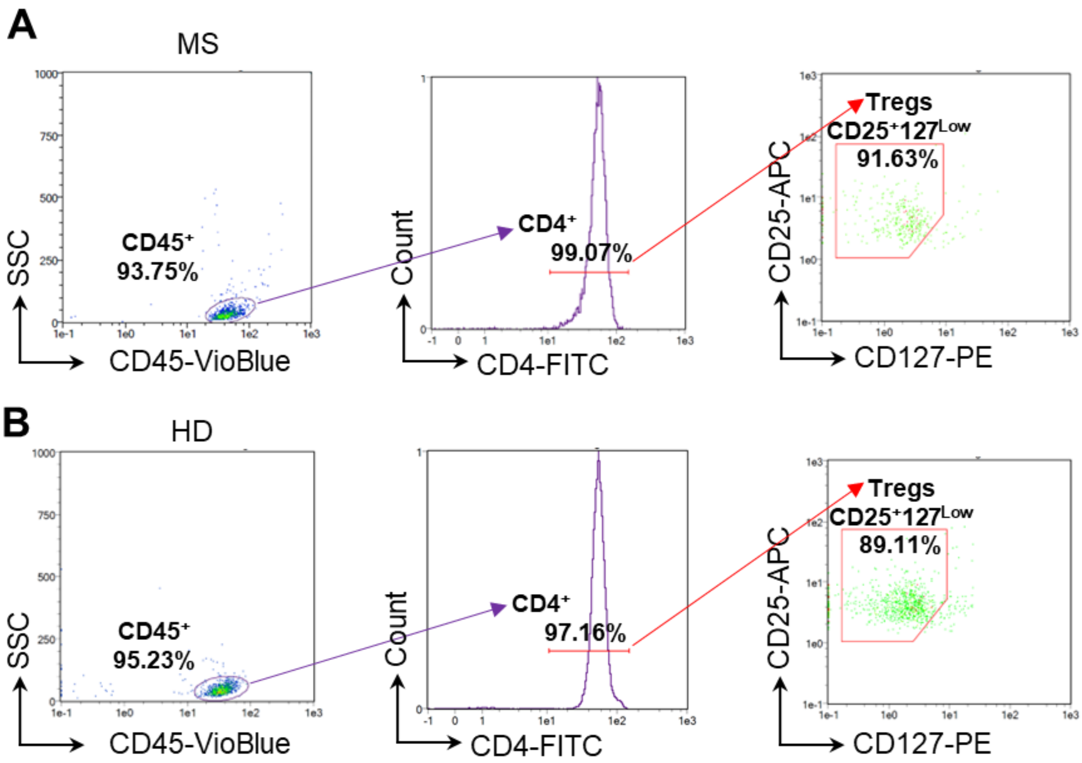

Figure S3. Gating strategy demonstrating the purity of isolated Tregs from (A) MS patients and (B) HDs. Tregs were isolated from peripheral blood using a $CD4^{+}CD25^{+}$ Regulatory T-Cell Isolation Kit II (130-091-301, Miltenyi Biotec, Bergisch Gladbach, Germany). Representative flow cytometry diagrams out of 8 are shown. A total of 4 patients were included in each of the MS and HD groups. The study was performed by cell labeling with antibodies from Treg Surface Marker Analysis Cocktail, anti-human (130-096-082, Miltenyi Biotec, Bergisch Gladbach, Germany) using flow cytometer MACSQuant Analyzer 10 (Miltenyi Biotec, Bergisch Gladbach, Germany).

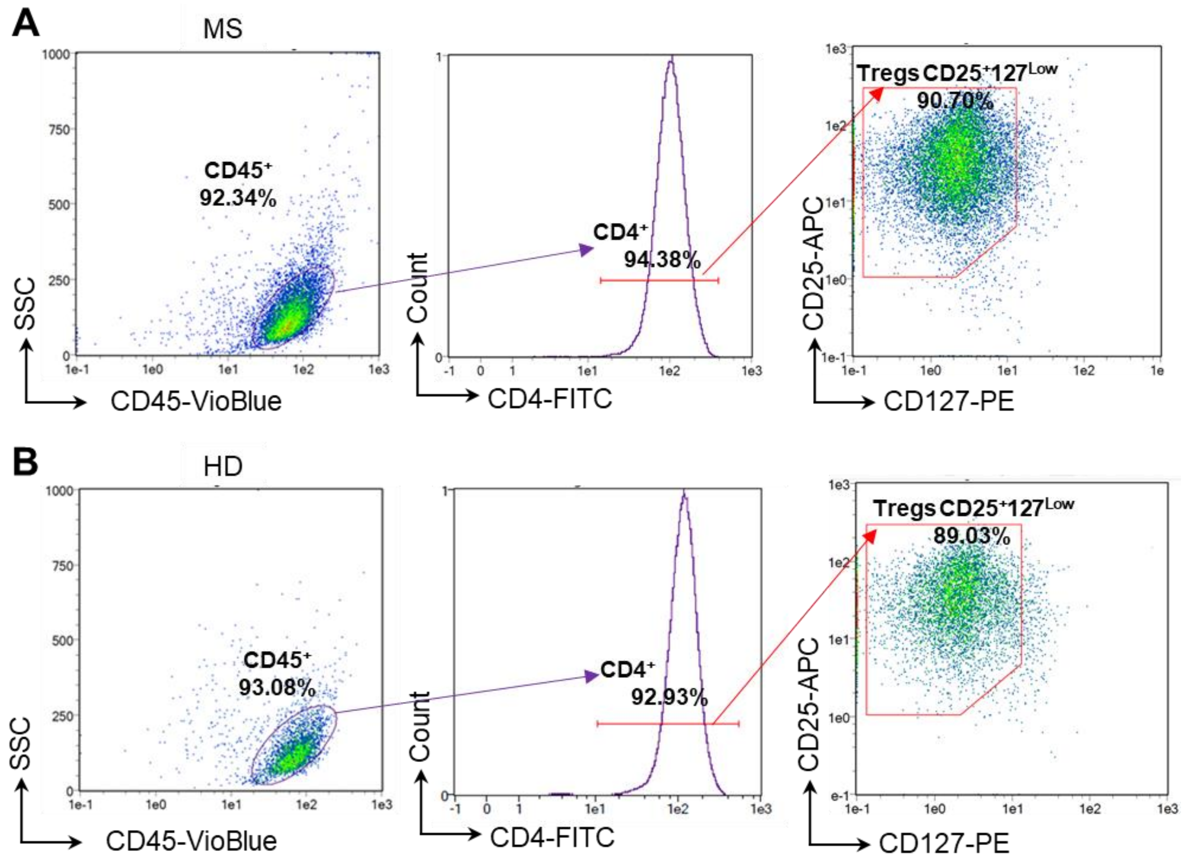

Figure S4. Gating strategy demonstrating the homogeneity of *ex vivo* multiplicated Tregs from (A) MS patients and (B) HDs for 15 days. Representative flow cytometry diagrams out of 8 are shown. A total of 4 patients were included in each of the MS and HD groups. The study was performed by cell labeling with antibodies from Treg Surface Marker Analysis Cocktail, anti-human (130-096-082, Miltenyi Biotec, Miltenyi Biotec, Bergisch Gladbach, Germany) using flow cytometer MACSQuant Analyzer 10 (Miltenyi Biotec, Bergisch Gladbach, Germany).

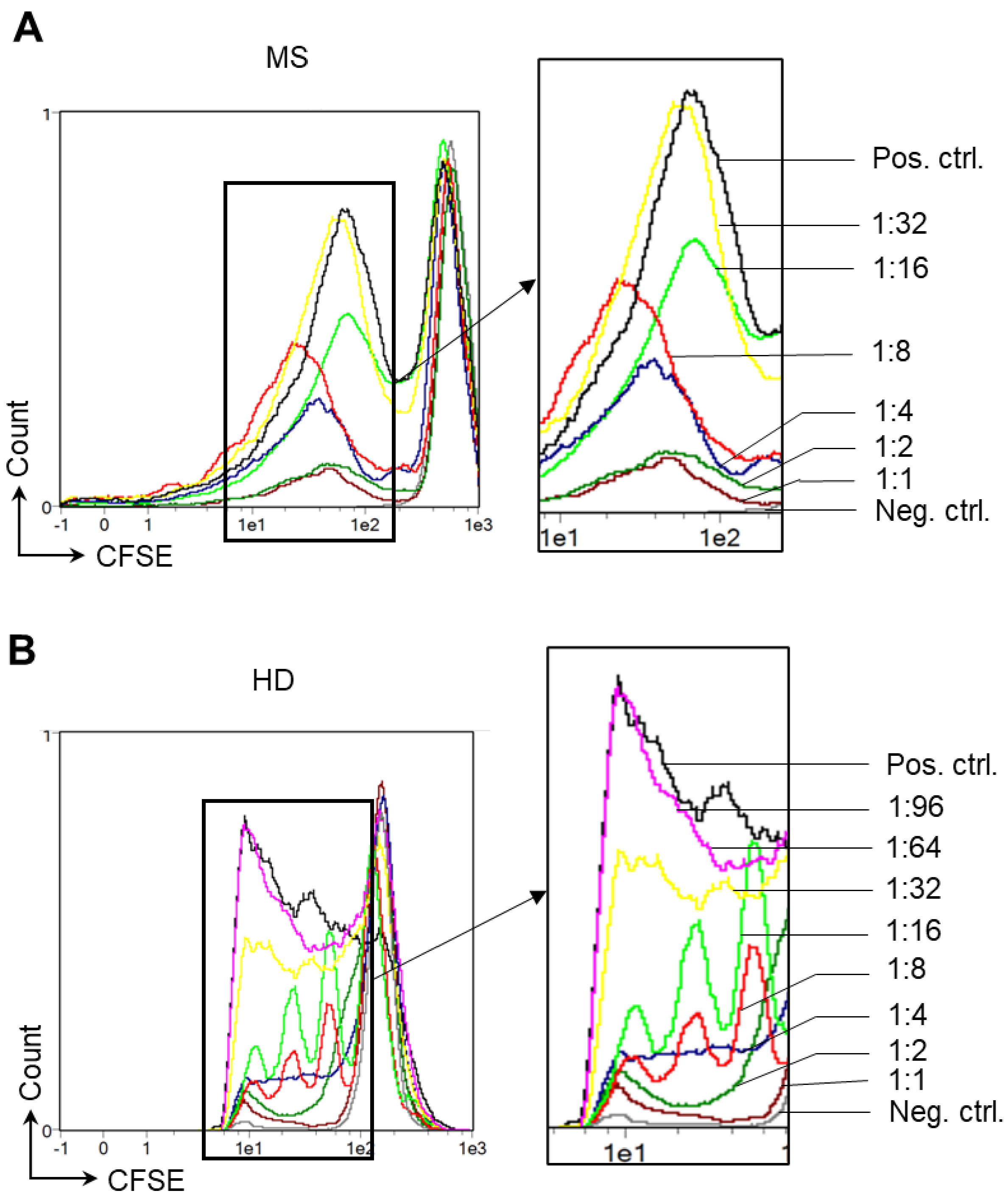

Figure S5. Representative proliferative diagrams of the mixed lymphocyte reaction study for *ex vivo* expanded Tregs isolated from (A) MS patients and (B) HDs. CFSE-labeled $CD4^+CD25^–$ responder cells were cocultured with accessory cells and Tregs at various ratios. The proliferation rate of target cells was monitored by reduction of the CFSE signal using flow cytometry. Neg. ctrl—negative control, $CD4^+CD25^-$ cells only. Pos. ctrl. — positive control, $CD4^+CD25^–$ cells cocultured with accessory cells.

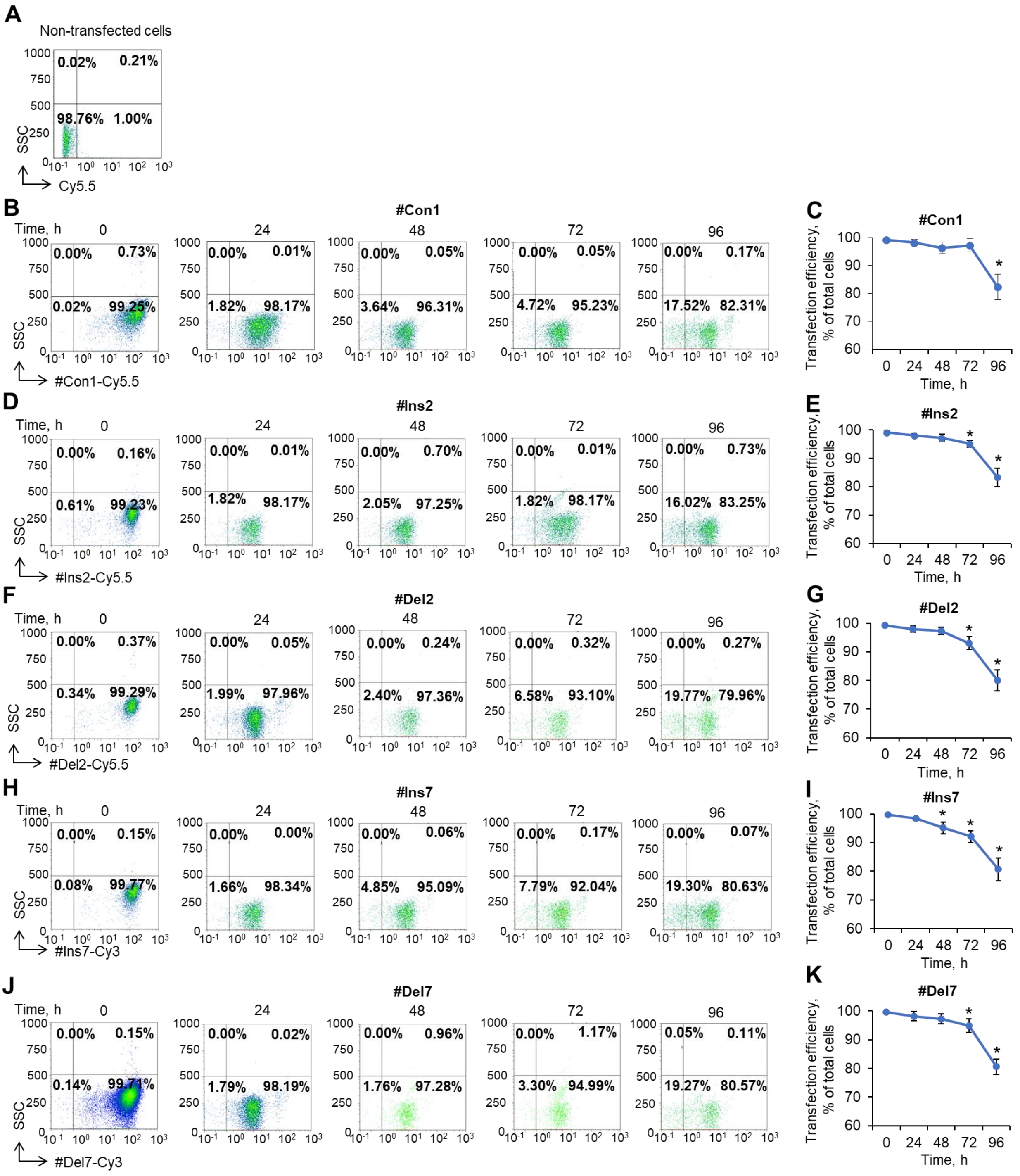

Figure S6. Transfection efficiency of Tregs with a single SSO targeting exon 2 or exon 7. Representative flow cytometry plots of cells transfected with Cy3- or Cy5.5-labeled oligonucleotides within 96 h posttransfection: (A) nontransfected cells; (B) cells transfected with control nonspecific oligonucleotide #Con1; (D) cells transfected with oligonucleotide #Ins2, which is capable of inducing insertion of exon 2; (F) cells transfected with oligonucleotide #Del2, which is capable of inducing deletion of exon 2; (H) cells transfected with oligonucleotide #Ins7, which is capable of inducing insertion of exon 7; (J) cells transfected with oligonucleotide #Del7, which is capable of inducing deletion of exon 7. (C, E, G, I, K) Transfection efficiency. The results are shown as the mean ± SD. N= 4. *$p \leq 0.05$ vs. cells at the 0 h time point by the Mann–Whitney U test.

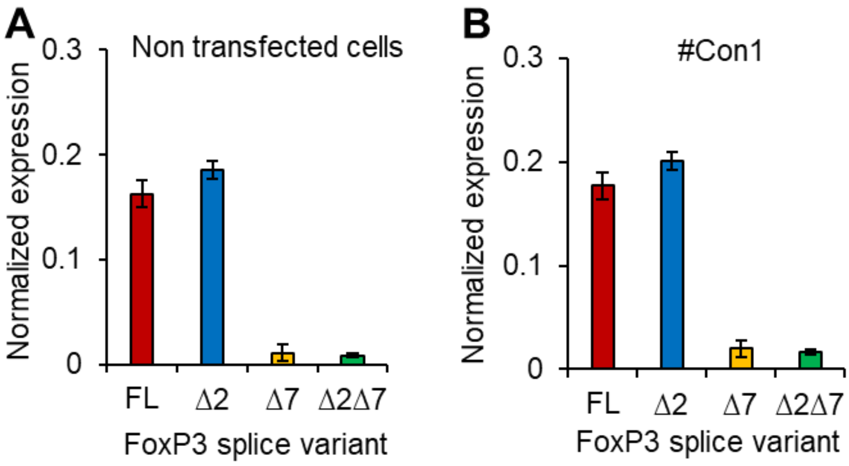

Figure S7. FoxP3 splice variant mRNA expression in (A) nontransfected Tregs and (B) Tregs transfected with the control nonspecific oligonucleotide #Con1 96 h after transfection. The levels of investigated mRNAs were normalized to the average expression of three reference genes: 18S, GAPDH, and beta-actin. N=4. The results are shown as the mean ± SD. FL, full-length splice variant; Δ2, splice variant with deleted exon 2; Δ7, splice variant with deleted exon 7; Δ2Δ7, splice variant with deleted both exon 2 and exon 7.

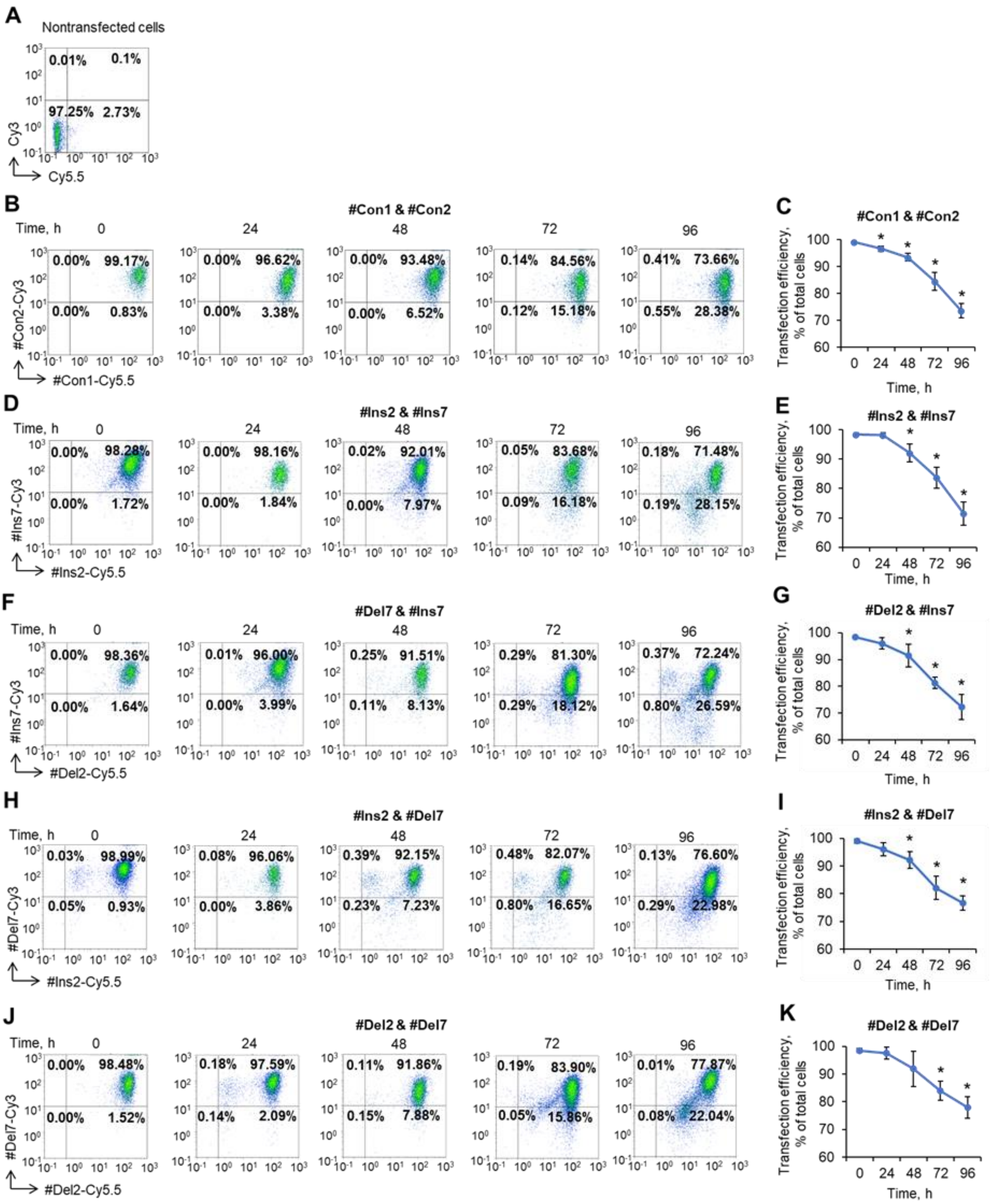

Figure S8. Transfection efficiency of Tregs with the couple SSOs targeting both exon 2 and exon 7. Representative flow cytometry plots of cells transfected with Cy3- or Cy5.5-labeled oligonucleotides within 96 h posttransfection: (A) nontransfected cells; (B) cells transfected with control nonspecific oligonucleotids #Con1 and #Con2; (D) cells transfected with oligonucleotides #Ins2, which is capable of inducing insertion of exon 2, and #Ins7, which is capable of inducing insertion of exon 7; (F) cells transfected with oligonucleotides #Del2, which is capable of inducing deletion of exon 2, and #Ins7, which is capable of inducing insertion of exon 7; (H) cells transfected with oligonucleotides #Ins2, which is capable of inducing insertion of exon 2, and #Del7, which is capable of inducing deletion of exon 7; (J) cells transfected with oligonucleotides #Del2, which is capable of inducing deletion of exon 2, and #Del7, which is capable of inducing deletion of exon 7. (C, E, G, I, K) Transfection efficiency. N= 4. The results are shown as the mean ± SD. N= 4. *$p \leq 0.05$ vs. cells at the 0 h time point by the Mann–Whitney U test.

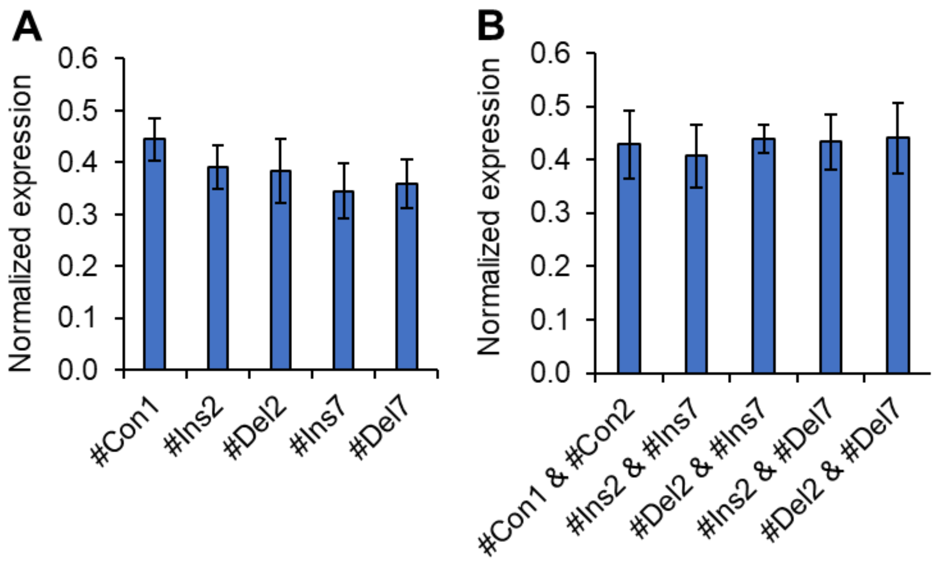

Figure S9. Total FoxP3 expression in Tregs transfected with SSO or control oligonucleotides. Tregs were transfected with (A) a single SSO targeting either exon 2 or exon 7 or (B) two SSOs targeting both exons. The mRNA levels were measured by real-time RT–PCR 96 h after transfection. mRNA levels were normalized to the average expression of three reference genes: 18S, GAPDH, and beta-actin. N=4. The results are shown as the mean ± SD.

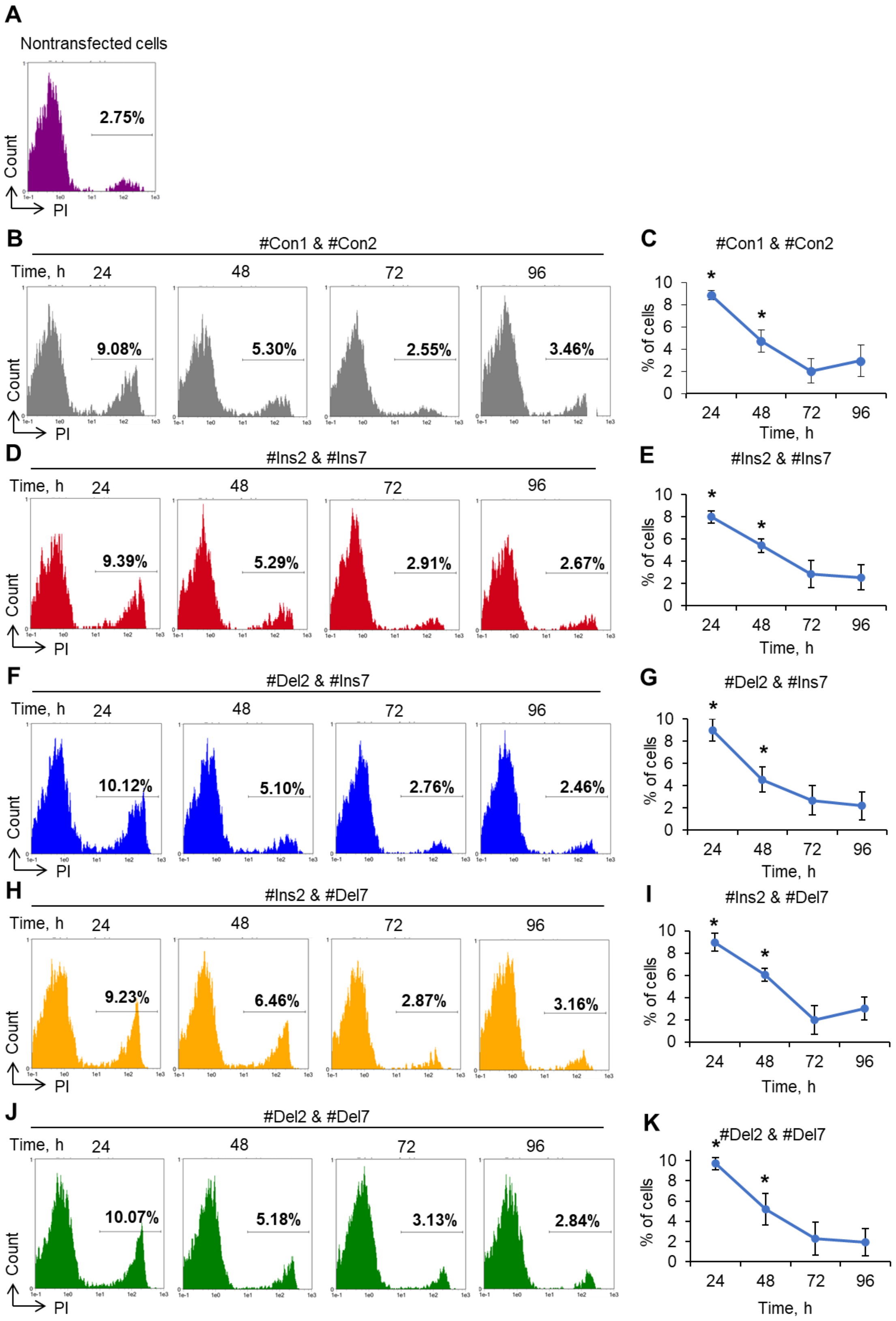

Figure S10. Monitoring of cell death by flow cytometry using Propidium Iodide (PI) staining. Representative flow cytometry plots of cells transfected with oligonucleotides within 96 h posttransfection: (A) nontransfected cells; (B) transfected with #Con1 & #Con2; (D) transfected with #Ins2 & #Ins7; (F) transfected with #Del2 & #Ins7; (H) transfected with #Ins2 & #Del7 (J) transfected with #Del2 & #Del7. (C, E, G, I, K) Percent of PI-positive cells obtained by flow cytometry. N= 4. *$p \leq 0.05$ vs. non-transfected cells by the Mann–Whitney U test.

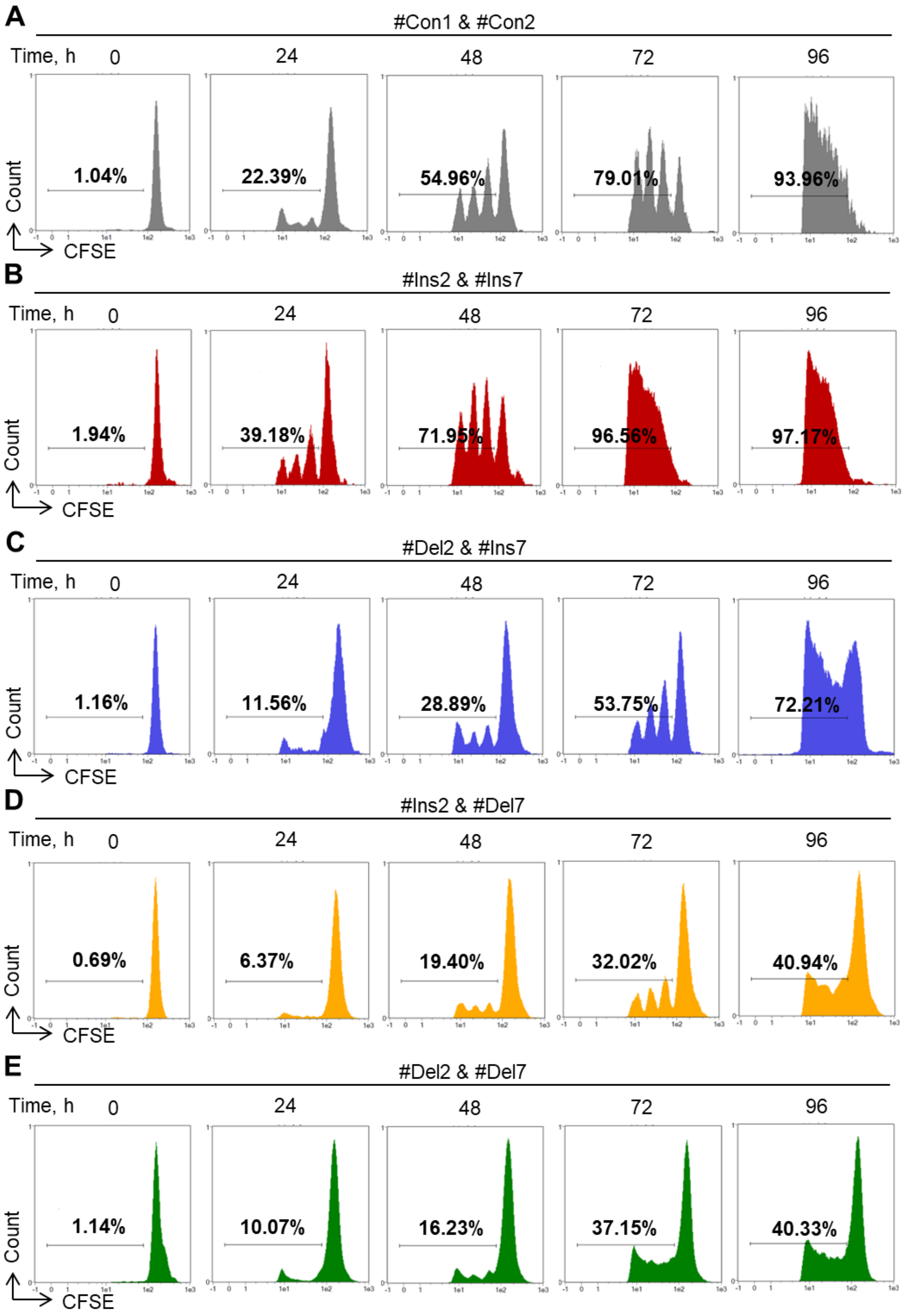

Figure S11. Proliferation of Tregs transfected with SSO. Representative flow cytometry diagrams of proliferating CFSE-labeled Tregs transfected with (A) control SSOs #Con1 & #Con2; (B) SSOs inducing full-length variant #Ins2 & #Ins7; (C) SSOs inducing Δ2 splice variant #Del2 & #Ins7; (D) SSOs inducing Δ7 splice variant #Ins2 & #Del7; and (E) SSOs inducing Δ2Δ7 splice variant #Del2 & #Del7. The percentage of total cells is shown. One representative example of a total of four is shown.

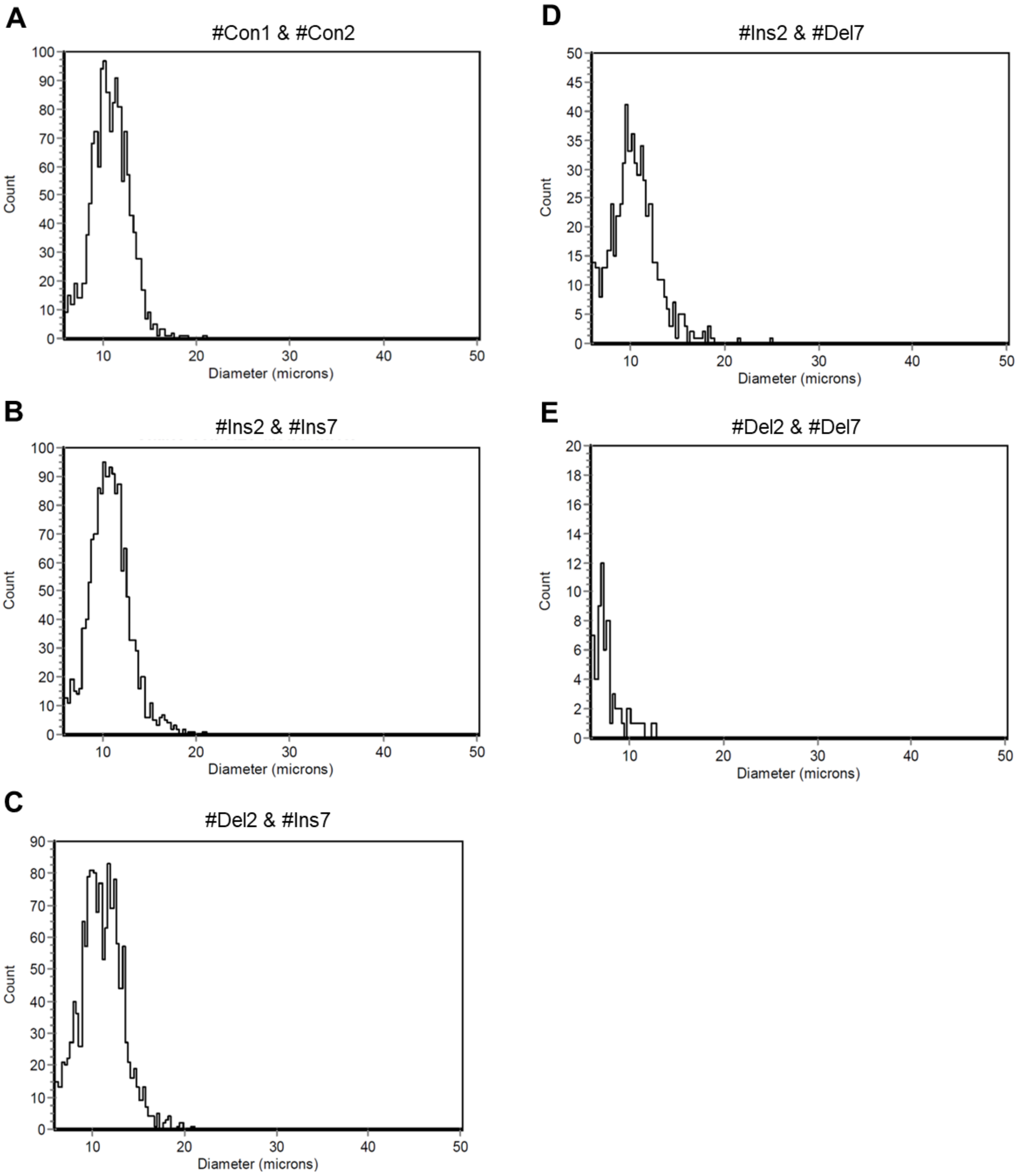

Figure S12. Size distribution of Tregs transfected with SSOs. The size distribution was analyzed by Cell Viability Analyzer Vi-Cell XR (Beckman Coulter, Brea, CA, USA) 96 h after transfection with (A) #Con1 & #Con2, (B) #Ins2 & #Ins7, (C) #Del2 & #Ins7, (D) #Ins2 & #Del7, and (E) #Ins2 & #Del7. Representative diagrams (total N = 4) created by Cell Viability Analyzer Vi-Cell XR software are shown.

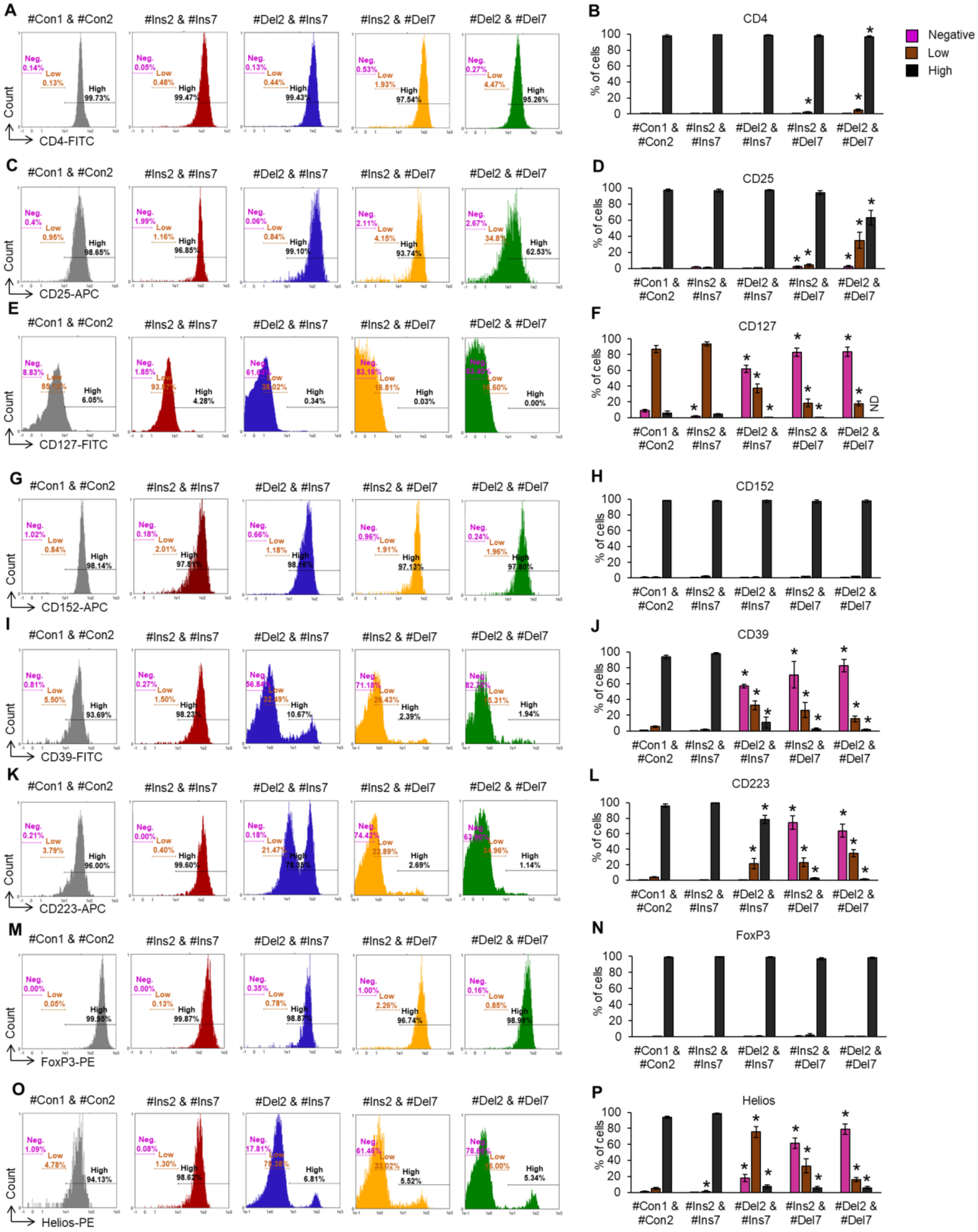

Figure S13. Flow cytometry study of Treg-associated cell markers. Tregs were transfected with SSOs and labeled with dye-conjugated antibodies for cell markers at 96 h post transfection. Representative flow cytometry diagrams demonstrating the proportion of cells with negative (Neg.), low and high expression of membrane surface cell markers (A) CD4, (C) CD25, (E) CD127, (G) CD127, (G) CD152, (I) CD39, (K) CD223 and nuclear transcription factor (M) FoxP3, (O) Helios. (B), (D), (F), (H), (J), (L), (N), and (P) The results of flow cytometry analysis revealing the proportion of cells with negative, low and high expression of cell markers. N= 4. *p ≤ 0.05 vs cells transfected with #Con1 & #Con2 oligonucleotides by Mann–Whitney U test. ND, not detected.

Table S4. Mean fluorescent intensities (MFI) of Treg-associated cell markers.

Transfection	Negative	Low	High
CD4			
#Con1 & #Con2	1.61	15.33	85.04
#Ins2 & #Ins7	1.34	15.32	203.86
#Del2 & #Ins7	1.50	16.70	172.86
#Ins2 & #Del7	1.41	13.45	191.08
#Del2 & #Del7	1.22	15.62	49.42
CD25			
#Con1 & #Con2	1.40	19.32	82.91
#Ins2 & #Ins7	1.04	12.78	195.21
#Del2 & #Ins7	1.21	18.07	176.47
#Ins2 & #Del7	1.11	15.70	112.95
#Del2 & #Del7	1.18	18.23	43.01
CD127			
#Con1 & #Con2	1.34	15.99	30.84
#Ins2 & #Ins7	1.22	10.52	39.68
#Del2 & #Ins7	1.10	18.55	70.54
#Ins2 & #Del7	1.28	16.28	35.46
#Del2 & #Del7	1.16	16.53	30.06
CD152			
#Con1 & #Con2	1.11	13.60	99.23
#Ins2 & #Ins7	1.30	18.46	134.40
#Del2 & #Ins7	1.22	17.56	87.76
#Ins2 & #Del7	1.21	16.79	103.93
#Del2 & #Del7	1.15	10.76	74.22
CD39			
#Con1 & #Con2	1.33	12.73	101.22
#Ins2 & #Ins7	1.20	15.07	140.99
#Del2 & #Ins7	1.04	10.22	100.49
#Ins2 & #Del7	1.25	13.53	77.27
#Del2 & #Del7	1.63	13.59	46.66
CD223			
#Con1 & #Con2	1.19	13.14	75.71
#Ins2 & #Ins7	1.22	11.73	212.60
#Del2 & #Ins7	1.33	14.06	83.46
#Ins2 & #Del7	1.26	19.79	69.51
#Del2 & #Del7	1.24	14.54	62.50
FoxP3			
#Con1 & #Con2	1.08	19.22	277.90
#Ins2 & #Ins7	1.17	16.94	317.26
#Del2 & #Ins7	1.15	17.89	121.1
#Ins2 & #Del7	1.04	15.16	139.41
#Del2 & #Del7	1.18	19.03	107.38
Helios			
#Con1 & #Con2	1.33	12.48	69.17
#Ins2 & #Ins7	1.20	19.90	116.72
#Del2 & #Ins7	0.85	13.98	135.65
#Ins2 & #Del7	0.92	10.12	97.73
#Del2 & #Del7	1.30	10.58	138.65

Mean from four independent measurements. Errors were in the range of ± 5% of the reported values.

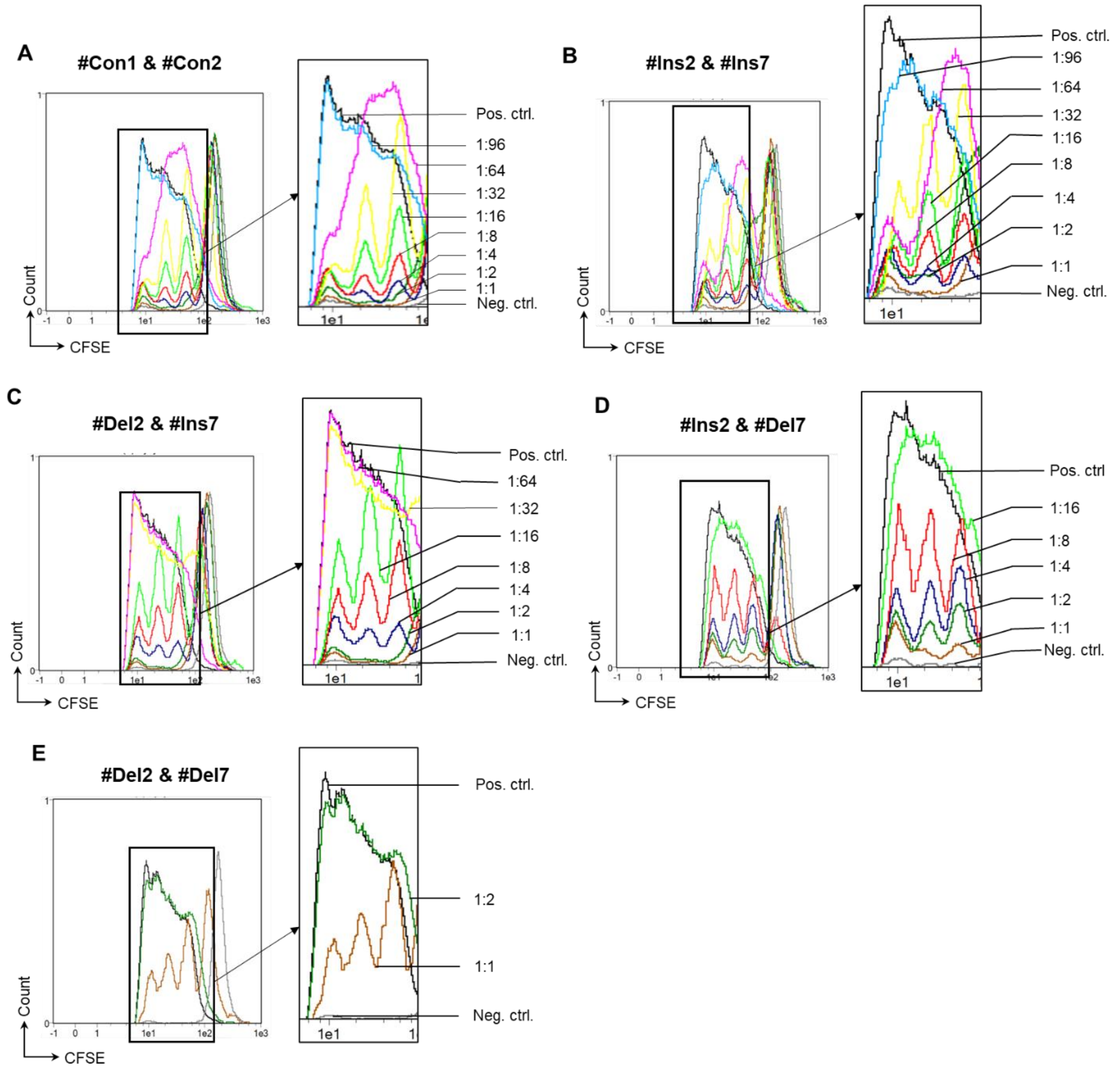

Figure S14. Representative proliferative diagrams of the mixed lymphocyte reaction study for transfected Tregs. (A) Cells transfected with control oligonucleotides #Con1 & #Con2; (B) with #Ins2 & #Ins7, (C) with #Del2 & #Ins7, (D) with #Ins2 & #Del7, and (E) with #Ins2 & #Del7. CFSE-labeled $CD4^{+}CD25^{-}$ responder cells were cocultured with accessory cells and transfected Tregs at various ratios. The proliferation rate of target cells was monitored by reduction of the CFSE signal using flow cytometry. Neg. ctrl—negative control, $CD4^{+}CD25^{-}$ cells only. Pos. ctrl. — positive control, $CD4^{+}CD25^{-}$ cells cocultured with accessory cells.

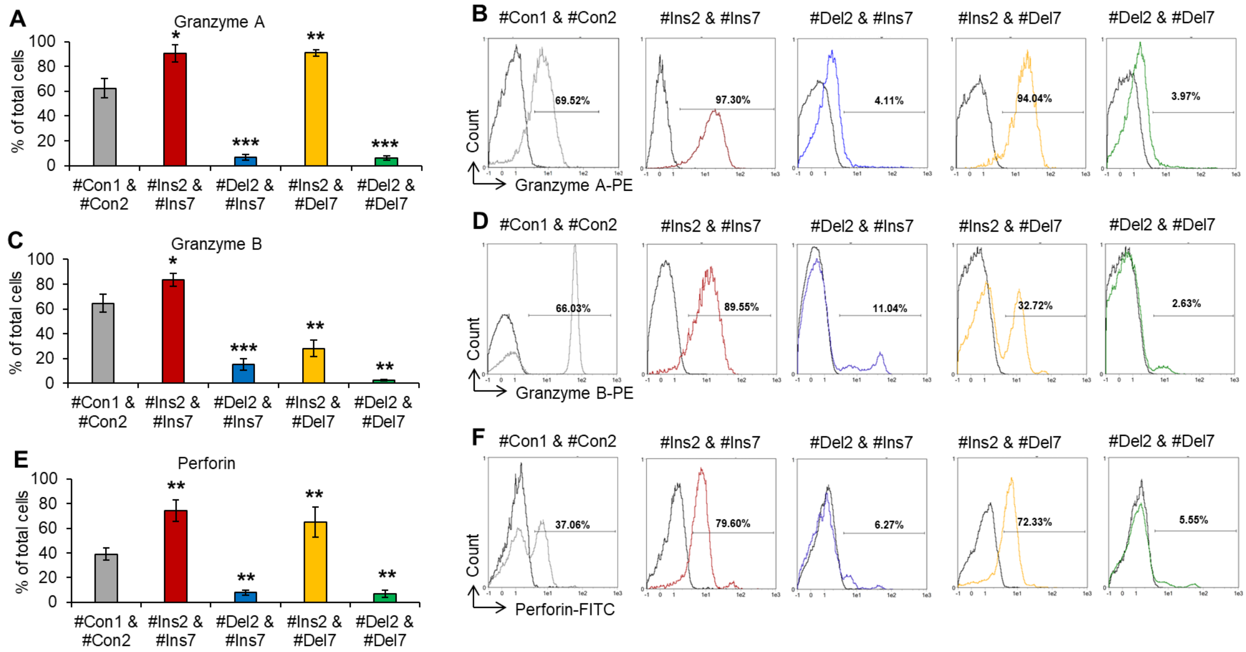

Figure S15. The ability of Tregs to produce apoptosis-inducing molecules: (A) granzyme A, (C) granzyme B, and (E) perforin. Transfected Tregs were incubated with stimulator and protein transport inhibitor or only with protein transport inhibitor as a control. Cells were labeled with CD4, CD25 and CD127 antibodies, fixed, permeabilized and incubated with antibodies against granzyme A or B or perforin. Levels of marker-positive cells were measured by flow cytometry. (B, D, F) Representative flow cytometry diagrams. Black lines indicate control cells incubated with protein transport inhibitor. Colored lines indicate stimulated cells incubated with protein transport inhibitor. N= 4. The results are presented as the mean ± SEM. *$p \le 0.05$; **$p \le 0.01$; ***$p \le 0.005$ vs cells transfected with #Con1 & #Con2 oligonucleotides by Mann–Whitney U test.
